# Ocular phenotypes in a mouse model of impaired glucocerebrosidase activity

**DOI:** 10.1038/s41598-021-85528-4

**Published:** 2021-03-16

**Authors:** Martin Weber, Sang-Won Min, Tom Truong, Jeffrey Hung, Stephanie Dale, Mike Reichelt, Savita Ubhayakar, Carol Cain-Hom, Miriam Baca, Zhiyu Jiang, Qingling Li, Robert Brendza, Han Lin, Chung Kung, William F. Forrest, Cristine Quiason-Huynh, Wendy Sandoval, Buyun Chen, Yuzhong Deng, Amy Easton, Oded Foreman, Abdoulaye Sene, Baris Bingol

**Affiliations:** 1 Departments of Neuroscience, Genentech Inc., 1 DNA Way, South San Francisco, CA 94080 USA; 2 Departments of Translational Immunology, Genentech Inc., 1 DNA Way, South San Francisco, CA 94080 USA; 3 Departments of Research Pathology, Genentech Inc., 1 DNA Way, South San Francisco, CA 94080 USA; 4 Departments of Drug Metabolism and Pharmacokinetics, Genentech Inc., 1 DNA Way, South San Francisco, CA 94080 USA; 5 Departments of Transgenic Technology, Genentech Inc., 1 DNA Way, South San Francisco, CA 94080 USA; 6 Departments of Microchemistry, Proteomics and Lipidomics, Genentech Inc., 1 DNA Way, South San Francisco, CA 94080 USA; 7 Departments of OMNI Bioinformatics, Genentech Inc., 1 DNA Way, South San Francisco, CA 94080 USA

**Keywords:** Diseases of the nervous system, Lipid-storage diseases, Parkinson's disease, Visual system

## Abstract

Mutations in the *GBA1* gene encoding glucocerebrosidase (GCase) are linked to Gaucher (GD) and Parkinson’s Disease (PD). Since some GD and PD patients develop ocular phenotypes, we determined whether ocular phenotypes might result from impaired GCase activity and the corresponding accumulation of glucosylceramide (GluCer) and glucosylsphingosine (GluSph) in the *Gba1*^*D409V/D409V*^ knock-in (*Gba* KI/KI; “KI”) mouse. *Gba* KI mice developed age-dependent pupil dilation deficits to an anti-muscarinic agent; histologically, the iris covered the anterior part of the lens with adhesions between the iris and the anterior surface of the lens (posterior synechia). This may prevent pupil dilation in general, beyond an un-responsiveness of the iris to anti-muscarinics. *Gba* KI mice displayed atrophy and pigment dispersion of the iris, and occlusion of the iridocorneal angle by pigment-laden cells, reminiscent of secondary open angle glaucoma. *Gba* KI mice showed progressive thinning of the retina consistent with retinal degeneration. GluSph levels were increased in the anterior and posterior segments of the eye, suggesting that accumulation of lipids in the eye may contribute to degeneration in this compartment. We conclude that the *Gba* KI model provides robust and reproducible eye phenotypes which may be used to test for efficacy and establish biomarkers for GBA1-related therapies.

## Introduction

Homozygous or compound heterozygous mutations in the *GBA1* gene encoding glucocerebrosidase (GCase) cause the lysosomal storage disorder Gaucher Disease (GD), while heterozygous *GBA1* mutations represent the most common genetic risk factor for Parkinson’s Disease (PD)^1–5^. Biochemically, these mutations lead to various levels of impairment in GCase activity, and correspondingly the accumulation of the lipid substrate glucosylceramide (GluCer) and its deacetylated metabolite glucosylsphingosine (GluSph) in the periphery and the CNS^6^. GD involves diverse pathological manifestations in liver, lung, spleen, and in some patients in the CNS^7–9^. Interestingly, pathological abnormalities in the eye and ocular dysfunction have been detected in patients with GD and in patients with other lysosomal storage diseases including Fabry disease, Dannon disease, and mucopolysaccharidoses^10–15^. Importantly, optical coherence tomography (OCT) imaging showed increased retinal thinning in heterozygous and homozygous *GBA* mutation carriers indicative of early-stage neurodegeneration in the eye^16^. Retinal thinning has also been detected in PD patients, and the severity of retinal thinning may correlate with both the Hoen and Yahr disease stage and dopaminergic neurodegeneration in these patients^17,18^. Taken together, these findings suggest that ocular pathology may offer a disease biomarker for GBA1-related disease and possibly for GBA1-related therapies.

The *Gba1*^*D409V/D409V*^ knock-in (*Gba* KI/KI) mouse model, referred to as *Gba* KI model hereafter, is characterized by a reduction in GCase activity and correspondingly the accumulation of the lipid metabolite, GluSph. Beyond this biochemical phenotype, this *Gba* KI mouse shows relatively mild phenotypic features, including some behavioral abnormalities as well as accumulation of proteinase K-resistant α-synuclein aggregates in the brain^19–21^. For example, Sardi et al.^19^ detected proteinase K-resistant α-synuclein immunoreactivity in only ~ 0.1% of the hippocampal area in 12 months-old *Gba* KI/KI mice. While there are reports of ocular phenotypes in mice bearing lysosomal gene mutations or deletions^22–24^, to the best of our knowledge, no ocular phenotypes have been reported in *Gba1*^*D409V/D409V*^ mice or any other mouse models of GD to date. Therefore, we set out to determine whether impaired GCase enzyme activity could lead to ocular pathology and dysfunction in *Gba* KI mice.

In the present study, we first sought to confirm the accumulation of GBA substrates in the CNS and peripheral organs of homozygous *Gba* KI/KI mice compared to wild-type (WT/WT, abbreviated as WT) littermates. Given the prior evidence of ocular phenotypes in GD patients, we then attempted to measure structural changes in the retina by OCT. However, we were unable to do so since the anti-muscarinic compound tropicamide, used to induce pupil dilation to enable OCT imaging, did not dilate the pupils in aged *Gba* KI mice. Interestingly, lack of pupil dilation was an age-dependent phenotype that progressively worsened in *Gba* KIs. To examine the structural correlates of this phenotype in *Gba* KI mice, we performed histology with Hematoxylin and Eosin (H & E) staining as well as electron microscopy of the eye, and found evidence of adherence of the iris to the lens (also known as posterior synechia), pigment dispersion from the iris, and accumulation of pigment-laden cells in the iridocorneal angle. Finally, we used Matrix-associated laser desorption ionization imaging mass spectrometry (MALDI-iMS) to determine whether lipids accumulate in the eye. Our results show that impaired GCase function is associated with accumulation of lipids in the eye, retinal thinning, and an age-dependent degeneration of several regions of the eye in KI mice. Thus, in contrast to the relatively mild pathological phenotypes reported to date^19,21^, this *Gba* KI mouse model shows pronounced ocular phenotypes. This can be used to determine the efficacy of GBA-related therapies in the eye and—since retinal thinning occurs in GD and PD—may provide a translatable biomarker for the clinical development of GBA-related therapies. More broadly, these data highlight the impact of lysosomal dysfunction on normal eye function.

## Materials and methods

The study was carried out in compliance with the ARRIVE guidelines (http://www.nc3rs.org.uk/page.asp?id=1357).

### Animals

All animal experiments were approved by the Genentech IACUC and comply with the Institute for Lab Animals’ guidelines for the humane care and use of laboratory animals. Animals were housed on a 14 h light/10 h dark cycle with ad libitum access to water and food. *Gba* KI mice were originally obtained from The Jackson Laboratory (C57BL/6 N-*Gba*^*tm1.1Mjff*^/J, strain number: 019106). These mice express mutant mouse D427V protein, homologous to the human D409V mutation. Their genetic background is C57BL/6, with a mix of C57BL/6 N and C57BL/6J. At Genentech the breeding colony was first expanded by crossings with C57BL/6N mice, in line with what was initially conducted at The Jackson Laboratory. Heterozygous matings generated both *Gba* KI/KI mice and *Gba* WT/WT littermate controls, also referred to as KI and WT, respectively. Since mutations for *Rd1, Rd8, Rd10* and *Rd12* were linked to retinal phenotypes^25–27^, mice were genotyped for the presence of these mutations (see “Genotyping” section). All mice were negative for mutations of *Rd1, Rd10* and *Rd12*, and all but four mice tested were homozygous mutants (MUT/MUT) for the *Rd8* mutation of the *Crb1* gene consistent with the presence of the *Rd8* mutation in the C57BL/6N background strain. The remaining four mice were confirmed as heterozygous (WT/MUT) for the *Rd8* mutation of the *Crb1* gene and were excluded from the analyses and the animal numbers stated in the paper, thereby ensuring homogeneity for *Rd8* between the experimental groups. Male mice were used throughout the study with the exception of the expanded pupil dilation measures, where male and female mice were used (see Supplements). The relevant n for each study is listed in Supplementary Table S1.

### Tissue collection

Mice for histology, Hematoxylin and Eosin (H & E) staining, and MALDI imaging were processed as follows: Mice from the different genotypes (*Gba* KI/KI vs. WT/WT littermates) and age groups were sacrificed in a pseudorandom order. Small groups of mice were deeply anesthetized with 2.5% Avertin (2,2,2-tribromoethanol, ~ 0.5 ml/mouse) and tail snips were collected. After transcardial perfusion with phosphate buffered saline, the superior part of the eye ball was marked with a grey marker to guide the orientation of the eye during sectioning. Eyeballs were then extracted along with the optic nerves. The right eye was stored in modified Davidson’s fixative solution (three parts 95% ethanol, two parts 10% neutral buffered formalin, one part glacial acetic acid, three parts distilled water) for 24 h at room temperature (RT) and then transferred into 70% ethanol solution (in distilled water) and kept at RT until sectioning and staining with H & E within days thereafter. The left eye was frozen on dry ice, and stored in a − 80 °C freezer until processing for MALDI imaging. Following removal of the eyes, the brain was extracted. The forebrain was subdissected and frozen for measures of lipids and GCase activity. For EM studies mice were anesthetized with Avertin, transcardially perfused with PBS followed by 4% EM grade paraformaldehyde (PFA). The eyes were extracted and placed into 4% EM grade PFA at 4 °C for 24 h.

### Genotyping for murine mutations linked to retinal degeneration phenotypes

Since passenger mutations would be in a position to confound phenotypes of genetically altered mice^28–31^, we genotyped our mice for murine mutations that have been linked to retinal pathology, namely mutations for *Rd1*, *Rd10*, *Rd12* as well as *Rd8* which is common in the C57BL/6N background strain^25–27^.

Genomic DNA was extracted from mouse tail biopsies using either the Agencourt DNAdvance Kit (Beckman Coulter) or the QIAamp Fast DNA Tissue Kit (Qiagen). Sections of the WT and mutated genes were made into gBlocks, synthetic, double-stranded DNA fragments, that served as controls for the PCR (Integrated DNA Technologies). The sequence of the gBlocks are listed in the supplements. The genomic DNA samples obtained were used in various reactions to determine the genotype at the following loci:

*Detection of Rd1 Mutation of the Pde6b gene* The *rd1* mutation is an insertion of an 8.5 kb section of the xenotropic murine leukemia virus (Xmv-28) into intron 1 of the Pde6b gene. A PCR was set up for detection of both wild type and mutant alleles with three primers^27^: Pde6brd1_F1 TACCCACCCTTCCTAATTTTTCTCAGC sits 5′ of the insertion, Pde6brd1_R TGACAATTACTCCTTTTCCCTCAGTCTG is the common reverse primer that sits 3′ of the insertion and Pde6brd1_F2 GTAAACAGCAAGAGGCTTTATTGGGAAC which sits in the 3′ end of the Xmv-28 insertion. For PCR amplification approximately 25 ng DNA were used in a 20 μL reaction volume containing 10 μL Type It Fast SNP PCR Mastermix (Qiagen), 0.8 μM each of the forward primers and 1.6 μM of the reverse primer for the combined *rd1* genotyping reaction. The wild-type (WT) amplicon is about 400 bp, while the mutant amplicon is about 550 bp.

*Detection of Rd8 Mutation of the Crb1 gene* DNA samples were amplified separately for WT and mutant *rd8* allele using primers as previously described^26^. Primer sequences included mCrb1 mF1: 5′-GTGAAGACAGCTACAGTTCTGATC-3′; mCrb1 mF2: 5′-GCCCCTGTTTGCATGGAGGAAACTTGGAAGACAGCTACAGTTCTTCTG-3′; and mCrb1 mR: 5′-GCCCCATTTGCACACTGATGAC-3′. For PCR amplification approximately 25 ng of genomic template DNA were used in a 20 μL reaction volume containing 10 μL Type It Fast SNP PCR Mastermix (Qiagen), 1.6 μM each of forward and reverse primer for the WT allele, and 0.8 μM of forward and 1.6 μM of reverse primer for the *rd8* mutant allele. Reactions initially were denatured at 94 °C for 5 min followed by 35 cycles at 94 °C for 30 s, 65 °C for 30 s, 72 °C for 30 s and a final extension at 72 °C for 7 min. Amplicons were separated using capillary electrophoresis and electropherograms were visualized using Genemapper 6 software (Thermo Fisher Scientific). Amplicon sizes are WT allele = 220 bp and *rd8* allele = 244 bp.

*Detection of Rd10 Mutation of the Pde6b gene* The *rd10* mutation is a single nucleic acid change in the Pde6b gene. The mutation was detected by a 5′ nuclease real-time PCR assay. The forward primer 5′-CAGCAAAGCCTATCGAAGAATCA-3′ and the reverse primer 5′-CATGGTCTGGGCTACATTGAAG-3′ (Integrated DNA Technologies) were used along with a WT probe labeled with 6FAM fluorescent tag on the 5′ end and an MGBNFQ quencher on the 3′ end, 5′-TACCACAACTGGCGC-3′. The mutant probe, 5′-CTACCACAACTGGTGC-3′ was labeled with a VIC fluorescent tag on the 5′ end and an MGBNFQ quencher on the 3′ end (Thermo Fisher Scientific). The 10 μL reaction was set up with a final concentration of 0.9 μM for each primer and 0.2 μM for each probe and 10 ng of genomic template DNA. Type It Fast SNP PCR Master Mix 2X (Qiagen) was used for extension of the primers. Thermal cycling conditions were 95.0 °C for 8 min, followed by 40 cycles of 95.0 °C (10 s), 62.0 °C (30 s) and 72.0 °C (10 s), respectively, using a real time PCR detection system.

*Detection of Rd12 Mutation of the Rpe65 gene* The rd12 mutation is a single nucleic acid change on the Rpe65 gene. We tested for this mutation via Sanger sequencing. PCR fragments were obtained using the forward primer 5′-TGACACCTAGTTTTAATATTTTGATCC-3′ and reverse primer 5′-CAGAGCTCTTGAACCCCATT-3′^27^.The same PCR setup and cycling conditions were used as for *rd10* mutation analysis, described above. The fragments were then sent to Quintara Biosciences for PCR cleanup and sequencing with the same primers that amplified the fragment. Sequencing results were obtained and aligned manually to determine genotype.

### GCase activity measures

Tissues were tested in a manner counterbalanced by genotype and age. GCase activity measures were conducted as previously described^32^. Mouse forebrain was homogenized in RIPA buffer (50 mM Tris, pH7.5, 150 mM NaCl, 0.5% Triton X-100). The resulting crude lysates were diluted with ten times of volume of assay buffer (150 mM citrate–phosphate buffer, pH 5.4, containing 0.25% w/w sodium taurocholate). After centrifugation at 4 °C, supernatant was collected and incubated with either 1 mM conduritol β-epoxide (CBE) or water for 1 h at RT, followed by incubation with 1 mM of 4-methylumbelliferyl-glucopyranoside (4MU-Glu) in assay buffer for 1 h at 37 °C. The reaction was terminated by adding half volume of 1 M glycine buffer (pH 12.5), and the cleaved 4-methylumbelliferone was measured by fluorimetry (Ex360/Em450). The values displayed are normalized to the mean value of 3 months old WT mice.

### Liquid chromatography–mass spectrometry/mass-spectrometry (LC–MS/MS)

Tissues were tested in a manner counterbalanced by genotype and age. Liver and brain samples were homogenized at 25 °C with a bead beater apparatus. The samples and calibration curves were pipetted out into low-binding extraction blocks followed by 5 μL of internal standard spiking solution. The samples were precipitated with 175 μL of methanol, vortexed and centrifuged. The supernatant was dried down and then reconstituted in 100 μL of initial starting mobile phase conditions for injection into the LC/MS/MS system. Since C18-glucosylsphingosine and C18-glucosylceramide are endogenous, C18-glucosylsphingosine-13C and C18-glucosylceramide-D5 were used as surrogate analytes for quantitation. Prior to sample analysis, parallelism experiments for both the unlabeled/labeled C18-sphingosines and unlabeled/labeled C18-ceramides were conducted to show that the surrogate analytes are a true representation of the endogenous levels. C18-galactosylsphingosine-D5 (Avanti Polar Lipids) and C18-galactosylceramide-D35 (Cayman Chemicals) were used as internal standards. Chromatographic separation was achieved with a HALO HILIC column with gradient elution using 25 mM ammonium formate (in 50:50 acetonitrile:water) and 5 mM ammonium formate (in 95:2.5:2.5 acetonitrile:water:methanol) with 0.2% formic acid. A tandem mass spectrometer with a turbo ion spray interface was used for LC–MS/MS analysis and was operated in positive ionization mode with multiple reaction monitoring at unit mass resolution for both Q1 and Q3 quadrupoles.

### Pupil dilation measures

Mice were tested in a manner counterbalanced by genotype and age to avoid confounding effects of time of day, or testing sequence. An observer blinded to genotype (and age) performed scorings. Mice were anesthetized with an intra-peritoneal (i.p.) dose of 70–80 mg/kg ketamine, and 15 mg/kg xylazine in sterile saline. Eyes were treated with a 1% solution of the anti-muscarinic pupil dilator tropicamide (Akorn). In a small fraction of recordings (4 mice in total) where sufficient anesthesia was not achieved with ketamine/xylazine, mice were briefly exposed to 4% of isoflurane in addition. After at least 4 min of Tropicamide exposure, mice were secured on an adjustable holding platform, a gel lubricant was applied to the eyes, and pictures of the anterior segment of the eye, which includes the limbus and the pupil, were taken using an imaging system. Pupil and limbus diameter measures were quantified from the same images by an experienced observer who was unaware of the experimental conditions. The limbus diameter was used as a proxy measure for the size of the eye. Pixels were used as the units of measurement. Ratios of the pupil diameter normalized to the limbus diameter were calculated too.

### Intra-ocular pressure (IOP) measures

Mice were tested in a manner counterbalanced by genotype and age. Young and aged male mice were anesthetized with an i.p. dose of 70–80 mg/kg ketamine, and 15 mg/kg xylazine in sterile saline. A rebound tonometer for rodents was used to measure IOP (Icare Tonolab). The probe of the tonometer was placed approximately 2 mm away from the center of the cornea at an angle perpendicular to the surface of the cornea. Six consecutive readings were obtained, the highest and lowest value were discarded and the average value from the remaining 4 values was noted. A total of three such averages were measured per eye in a manner that altered between the right and the left eye. The average across all six of these average values (3 per eye) was calculated. Pressure was measured in mmHg.

### Histology

Following fixation, eyes were transferred into individual tissue cassettes and were processed with a tissue processor starting in 70% ethanol, followed by dehydration in alcohols, clearing in xylene, and infiltration with Surgipath Paraplast (Leica Biosystems). A tissue embedder was used to embed the eyes sagittally in metal base molds. The marked superior side of the eye was orientated so that it was facing a side of the metal base mold with the optic nerve towards the cassette label. 4 µm tissue sections were cut with an automated microtome at the level of the retina-optic nerve junction. The water bath was set to 40 °C and sections were collected on Superfrost Plus (ThermoFisher Scientific) charged glass slides. Sections were allowed to dry on slides overnight and baked in a 70 °C oven for 20 min before staining. Slides were stained in H & E and coverslipped with xylene-based mounting media.

Histological procedures for MALDI imaging experiments are described in the “Methods” section.

### Quantification of histological data

Samples were quantified in a manner counterbalanced by genotype and age and data processing or scorings were conducted blinded to genotype and age. Bright field images from histological H & E sections of the eye ball were acquired by an automated slide-scanning platform at a final magnification of 200 ×. These cross-sectional pictures were used to quantify the distance (in µm) between the opposing parts of the sphincter muscles that surround the iris. Since this measure only corresponds to the pupil diameter if the histological cross section goes perfectly through the center of the pupil, this measure is more accurately labeled as pupil secant length and serves as a proxy measure for the size of the pupil diameter. For example, pupil secant length can be zero when the pupil is entirely closed, or when a very narrow pupil is not perfectly aligned with the histological plain. Correspondingly, the maximal distance between opposing ends of the lens (in µm) as apparent in the cross-section (lens secant length) was used as a proxy measure for the size of the eye. The presence or absence of posterior synechia, pigment-laden cells in proximity to the iridocorneal angle and cataracts of the lens were determined by an experienced pathologist. A binary (yes/no) scale was used to score these data. Besides, to illustrate the severity of iris pathology, an aggregate score consisting of individual iris-related binary scores was established. The binary scores were the presence (score = 1) or absence (score = 0) of “stromal atrophy”, “pigment dispersion from the iris”, “accumulation of pigment-laden cells in the iridocorneal angle”, and “posterior synechia”. These four individual scores were summed up yielding a scale from 0 to 4, with higher scores representing more severe iris pathology.

Thickness of the entire retina and its layers was quantified with a Matlab software package (MathWorks). Since the H & E tissue sections were oriented to intersect with the optic disc, an upper (i.e. superior to the optic disc on the image) and a lower section of the retina (i.e. inferior to the optic disc on the image) were defined by separate regions of interest (ROIs). The ROIs excluded the optic disc area due to the absence of retinal layers in this region. Correspondingly, the very peripheral ends of the retina near the ora serrata where retinal tissue first thins out before it is ultimately absent were also excluded from the ROIs. The two ROIs were aggregated for the purpose of data analysis. Within the ROI borders, the different retinal layers, including the ganglion cell layer (GCL), the retinal nerve fiber layer (RNFL), the inner plexiform layer (IPL), the inner nuclear layer (INL), the outer plexiform layer (OPL), the outer nuclear layer (ONL), and the rod and cone segment layer (RCSL) were identified by intensity thresholding and simple morphological filtering. Total retinal area was defined as all retinal layers in between the RCSL and the inner limiting membrane (ILM), including both of these layers. Area measures of the retina and its layers were calculated and normalized relative to the perimeter length of the retina, which was determined by the length of the inner limiting membrane (ILM), i.e. the retinal tissue closest to the lens. Normalizing area measures by dividing by this perimeter line (= width) thus provided a proxy measure of the layer thickness in µm.

### Matrix-assisted laser desorption ionization imaging mass spectrometry (MALDI-IMS)

MALDI-IMS^33^ methods were modified from previous reports^34^. Eyes were sectioned on a cryomicrotome. The 12 µm thick sections were collected on adhesive tape windows (Leica Biosystems), dehydrated for 1 h, and mounted onto indium tin oxide coated glass slides (Bruker Daltonics) via double-sided tape. The Matrix solution (2,5-dihydroxybenzoic acid matrix (40 mg/mL, 70/30 methanol/H_2_O, 0.2% trifluoroacetic acid; 2 µM internal standard spike matrix) was applied to the sections with an automated sprayer. Eight passes of the sprayer nozzle were employed using intermittent dry times of 0.5 s. Line spacing was 3 mm and the nozzle temperature was 75 °C. MALDI MS imaging analysis used a 7.0 T SolariX – XR FT-ICR system (Bruker Daltonics) with a dual ESI-MALDI source. Broadband MS spectra were acquired in positive ion mode using a mass range of m/z 150–1000. Pixel resolution was 40 µm. Laser settings were optimized for the internal standard response. A 95% data reduction was used and the internal standard lock mass was m/z 401.072117. The extraction of analyte images was guided by the exact mass of each compound (C18-GluSph = m/z 462.342592; C18-GluCerNa = m/z 750.585439; phosphatidylcholine (Pc 32:0) = m/z 734.569432; phosphatidylcholine (PC; 34:1) = m/z 760.585082; phosphatidylcholine (PC 36:1)Na = m/z 810.598326; phosphatidylcholine (PC 38:6)Na = m/z 828.55). FlexImaging software (Bruker Daltonics; mass tolerance: ± 2 mDA) was used for image display. Eye sections were subsequently stained by H & E to enable anatomical verification of the MALDI images.

### MALDI-IMS data analysis

Four ROIs were identified by a pathologist blind to the experimental condition based on visual inspection of the H & E sections. ROIs included the entire eye ball, and two subregions consisting of the retina as well as a crescent-shaped area with the corners of the crescent at the ciliary bodies and included large sections of the anterior and posterior chambers as well as the iris. A fourth, residual, ROI was calculated by subtracting the area and intensity data of these two sub regions from that of the entire eyeball. This residual ROI mostly consists of large portions of the lens and the posterior chamber. H & E images were then manually co-registered with MALDI images by an observer unaware of the experimental conditions. Average intensities per pixel for the GluSph, GluCerNa, PC (32:1); PC (34:1), PC (36:1)Na, and PC (38:6)Na signal were then extracted from each of the ROIs for each mouse and normalized to the internal standard. In order to express GluSph and GluCer values relative to control PC lipids (PC (32:0), PC (34:1), PC (36:1)Na, and PC (38:6)Na), an aggregate abundance value was calculated for each tissue section by measuring the fold-change in abundance of each PC lipid relative to the control young (3 months) WT group, and then by obtaining the geometric mean of these fold changes across the four PC lipids. The “aggregated PC abundance” changes were then used to normalize the abundance of GluSph and GluCer in each tissue section.

### Back-scattered electron scanning electron microscopy (BSE-SEM)^35^

BSE-SEM procedures were conducted as previously described by our laboratories^35^: Eyes were dissected and stored for 24 h in 4% EM grade PFA at 4 °C, transferred into modified Karnovski fixative (2.5% paraformaldehyde and 2% glutaraldehyde in 0.1 M cacodylate buffer, pH 7.2) for ≥ 24 h at 4 °C, washed with EM-grade water and post-fixed with 2% aqueous osmium tetroxide for 4 h. Tissues were again washed again in EM-grade water, stained overnight “en block” with 1% (w/v) uranyl acetate at 4 °C, dehydrated with ascending ethanol concentrations, rinsed two times with propylene oxide and embedded in epoxy resin. Semithin sections (500 nm thick) were cut with a diamond knife on an ultramicrotome, transferred to carbon-coated histology glass slides, dried, stained with 4% aqueous uranyl acetate for 15 min and 0.1% Reynold’s lead citrate for 1 min, rinsed with EM-grade water, and dried again. SEM was conducted on a GeminiSEM 300 with a field emission gun in “high current mode”, using SmartSEM software (Carl Zeiss). Imaging was typically conducted with the backscatter electron detector at 8.5 mm working distance, 30 µm (standard) aperture, and 3–6 keV acceleration voltage, noise reduction via line averaging, using an image size of at least 4096 × 3072 (4 k × 3 k) pixels. For pictures of ultrastructural details, pixel sizes between two and five nm were used. Greyscale inversion was applied to obtain transmission electron microscopy-like images. The contrast and brightness of whole images was adjusted, regions of interests were cropped, and 300 dots per inch (dpi) print resolution for figure preparation was achieved with Photoshop CS4 (Adobe).

### Experimental design and statistical analysis

A cohort of male young and aged *Gba* WT and KI mice was used throughout the main study, beginning with IOP and pupil dilation measures when the young and aged mice were approximately 2.2 months and 16.6 months of age, respectively. Tissues were harvested from these mice when they were 3 and 17-months old (~ 2.7 and 17.2 months old on average), respectively, and their tissues were used for post-mortem studies (GCase activity, lipid measures via LCMS, pupil secant length, pigment-laden cells close to iridocorneal angle, posterior synechia, cataracts, retinal thickness). Since MALDI imaging has low throughput, eyes from a subset of 23 mice (n = 5 to 6 per group) were selected from this cohort, ensuring that the mice from the different genotypes were a proper sub-cohort of litter-mates. IOP, pupil dilation, and all of these post-mortem studies therefore had a 2-factorial between-subjects study design with the factors genotype and age. All parametric data were analyzed with ANOVAs followed by post-hoc comparisons using Tukey’s HSD to account for more than two comparisons between factors or factor levels. In cases where binary scoring data was analyzed and factorial logistic regression analysis was not feasible due to the relative lack of variation within cells (binary data for posterior synechia, pigment-laden cells in proximity to the iridocorneal angle, cataracts), Fisher’s exact test was calculated across all cells (i.e. young *Gba* WT, young *Gba* KI, aged *Gba* WT, aged *Gba* KI) or as post-hoc tests for specific comparisons between individual groups to test for differences between groups. Similarly, for the iris pathology aggregate scale which is based on the combination of individual binary scores, a Fisher’s exact test was calculated across all cells based on four experimental groups (young *Gba* WT, young *Gba* KI, aged *Gba* WT, aged *Gba* KI) and five score levels (0, 1, 2, 3, 4).

Three male mice (2 KI/KI, 1 WT/WT, 19.1 to 19.8 months old at tissue harvest) were used for EM experiments. An expanded pupil dilation study with mice from additional cohorts, including mice of various ages as well as female mice, is described in the supplements. Line or bar graphs are shown with mean ± SEM values. A 2-tailed alpha of 0.05 was used. *p < 0.05, **p < 0.01, ***p < 0.001.

All results of the statistical tests are listed in Supplementary Table S1.

## Results

### Reduced GCase activity and accumulation of substrates in *Gba* KI mice

**Figure 1:**
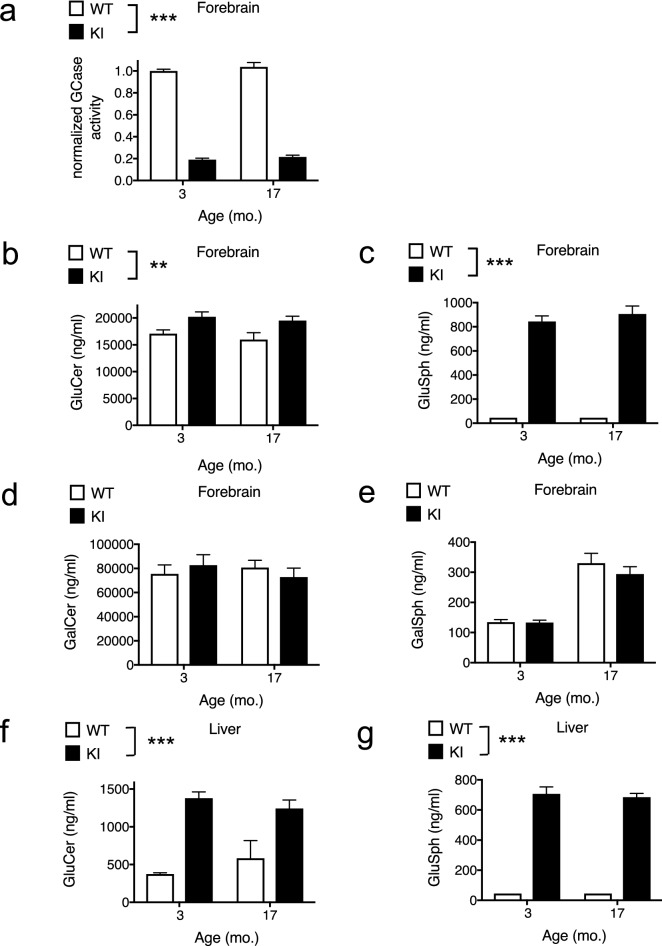
Decreased GCase activity and increased GluSph and GluCer levels in *Gba* KI mice. (**a**) GCase activity in the 4MU-Glu assay. GluCer levels (**b**) and GluSph levels in forebrain (**c**). GalSph levels (**d**) and GalCer levels (**e**) in forebrain. GluCer levels (**f**) and GluSph levels in liver (**g**). GalSph and GalCer levels in the liver were below detection limits (not shown). GCase activity was normalized to average GCase activity from 3-months old WT mice. Asterisks denoting significance levels for main effects of genotype are indicated next to the genotype symbols above the graphs. **p < 0.01, ***p < 0.001. Values are displayed as mean ± SEM.

GCase activity as measured by the turnover of 4MU-Glu in forebrain tissue lysates was ~ fivefold lower in *Gba* KI than in WT mice (Fig. 1a; *p* < 0.0001). This decrease was detected at 3 months of age and did not progress further in 17 months old mice. These data confirm GCase activity impairment in *Gba* KI mice in line with published reports^19,36^.

Since *Gba* KI mice show reduced GBA activity, we next measured the levels of GCase substrates in the CNS. Mirroring the reduction in GCase activity, its substrate GluCer was increased in the forebrain by ~ 1.2 fold in *Gba* KI mice as compared to WT mice across the age groups (Fig. 1b; *p* = 0.0018). The levels of GluSph, a deacetylated metabolite of GluCer^37^, was substantially increased in *Gba* KI mice relative to WT mice (~ 20-fold) in all age groups (Fig. 1c; *p* < 0.0001). The abundance of GalCer and GalSph did not change between genotypes (Fig. 1d, e, *p* ≥  0.38, each), but GalSph levels were increased by > twofold with age in both genotypes (Fig. 1e, *p* < 0.0001). Similar genotype-dependent changes in substrate levels for GluCer and GluSph were observed in the liver (Fig. 1f, g; *p* < 0.0001, each). Thus, reduced GCase activity in the *Gba1*^*D409V/D409V*^ mice is associated with specific accumulation of GCase substrate and metabolites.

### Assessing *Gba* KI and WT mice for mutations linked to retinal pathology

Since mutations for *Rd1*, *Rd8*, *Rd10* and *Rd12*, have been linked to retinal pathology in mice, we genotyped our mice for these mutations to assess if the phenotypes in our study could possibly be due to any of these mutations acting as passenger mutation of the *Gba* knock-in^28–31^. All mice were wild-types for *Rd1*, *Rd10* and *Rd12*. All but four mice were homozygous mutants for *Rd8*, consistent with the presence of the *Rd8* mutation in the C57BL/6N background strain^25–27^. The four mice that were heterozygous for *Rd8* were excluded from all analyses to ensure homogeneity between experimental groups with respect to *Rd8*. Thus, the *Gba* WT and KI mice of the present study were identical with respect to their genotype for all of these RD mutations showing that these RD mutations do not act as a passenger mutation of the *Gba* KI.

### Pupil dilation deficits in response to the anti-muscarinic compound tropicamide in aged *Gba* KI mice

**Figure 2:**
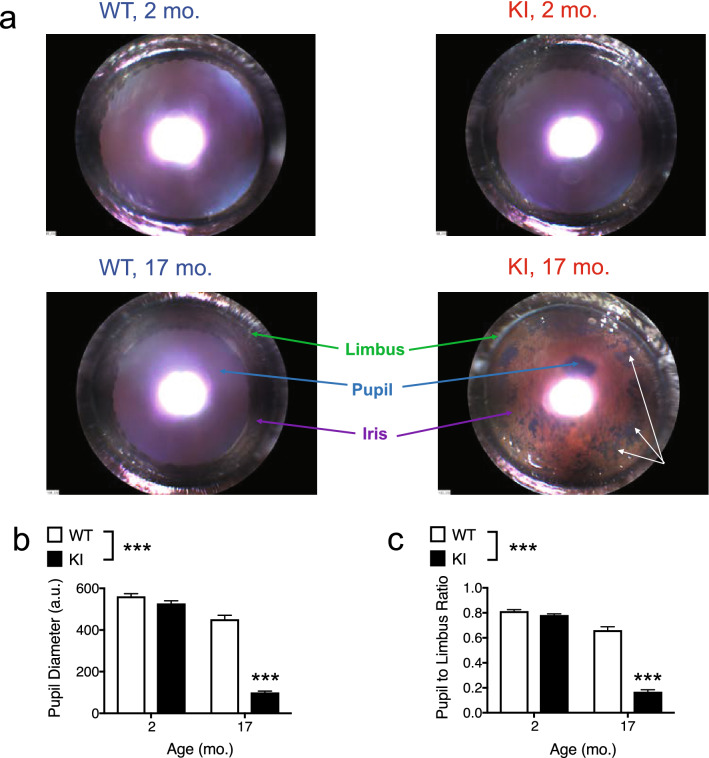
Pupil dilation deficits in response to tropicamide in aged *Gba* KI mice. (**a**) Representative images of the anterior segment in anesthetized 2- versus 17-months old *Gba* KI and WT mice following tropicamide treatment. The bright spot in the middle area of the images are reflections of the axial light illumination. The arrows indicate cracks and holes in the iris of a 17-months old KI mouse. (**b**) Pupil diameter following tropicamide treatment measured from (**a**). (**c**) Pupil to limbus diameter ratio measures were calculated to normalize pupil measures relative to limbus diameter, a proxy measure fore eye size. a.u. arbitrary units. Asterisks denoting significance levels for main effects of genotype are indicated next to the genotype symbols above the graphs; those for post-hoc tests between individual genotype- and age-combinations are indicated within the graphs. Main effects of age are not indicated. ***p < 0.001. Values are mean ± SEM.

Since retinal thinning has been detected in *GBA* mutation carriers and in patients with GD or PD^16–18^, we wanted to measure retinal thickness in *Gba* KI mice. When we attempted to perform OCT studies to assess retinal thickness in aged *Gba* KI mice, however, we were unable to do so since the pupils of aged *Gba* KI mice did not dilate in response to tropicamide, a reagent used to induce pupil dilation for access to the retina for imaging by OCT. To document this observation systematically, we applied tropicamide, measured pupil as well as limbus diameter, a proxy measure of eye size, in ~ 2 and 17-months old *Gba* KI and WT mice, and calculated pupil to limbus diameters ratios (Fig. 2a–c).

*Gba* KI mice showed age-dependent reduction in pupil diameter when compared to WT mice (*p* < 0.0001). While the two genotypes had similar pupil dimeters at 2-months, *Gba* KIs had a 4.5-fold reduction in pupil diameter at 17 months (Fig. 2b). This reduction in pupil size in aged KIs was not due to an overall change in eye size since normalization of pupil diameter with limbus diameter (pupil to limbus ratios) confirmed the reduced pupil size in *Gba* KIs at 17-months (Fig. 2c). In addition, damage to the iris, likely consisting of cracks and holes was apparent in aged *Gba* KI mice (Fig. 2a, white arrowheads). Longitudinal studies in a separate cohort of animals showed progressive reductions in pupil diameter with a near linear trajectory in *Gba* KI mice (Supplementary Figure S1, Supplementary Table S1). Besides there was a main effect of genotype with smaller pupils in KI mice, and aged mice had smaller pupils when compared to young mice (*p* < 0.0001, each).

### Pupil dilation deficits in aged *Gba* KI mice are apparent post-mortem

**Figure 3:**
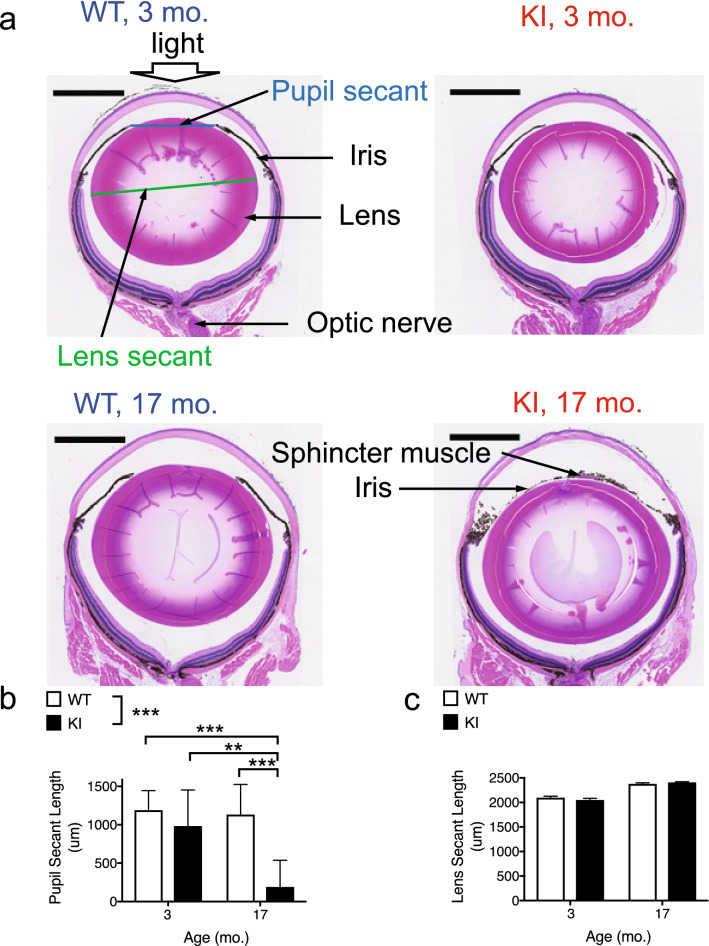
Pupil dilation deficits in aged *Gba* KI mice are apparent post mortem. (**a**) Representative images taken from sagittal cross sections through the globe at the level of the optic nerve in 3- versus 17-months old *Gba* KI and WT mice. The picture to the upper left illustrates the path of the light into the eye (from top to bottom), and indicates how pupil secant length and lens secant length, proxy measures for the size of the pupil and the lens, respectively, were derived from the histological sections. In the picture of the aged KI mouse (bottom right) the sphincter muscle of the iris is visible, indicating that the cross section is in close proximity to the center of the pupil, but no pupil opening is apparent. This indicates that the pupil is either completely closed, or that the center of pupil is not perfectly located in this histological plain, and that the size of the pupil opening is so small that a pupil secant cannot be detected in this plain. Pupil secant length (**b**). Lens secant length (**c**). The scale bar denotes 1000 µm. Asterisks denoting significance levels for main effects of genotype are indicated next to the genotype symbols above the graphs; those for post-hoc tests between individual genotype- and age-combinations are indicated within the graphs. Main effects of age are not indicated. *p < 0.05, **p < 0.01, ***p < 0.001. Values are mean ± SEM.

To examine if the pupil dilation defects indicate a specific unresponsiveness to pupil dilators such as tropicamide or highlight a broader ocular dysfunction, we measured pupil diameter in dissected eyes in the absence of prior treatment with pupil dilators (Fig. 3a). Quantification of the apparent pupil size on pictures from transverse cross-sections through the midline of the eye (pupil secant length, blue line) in 3- versus 17-months old mice indicated that KI mice had age-dependent reductions in pupil size when compared to WT mice (Fig. 3b, *p* = 0.0063).This yielded to more than fivefold smaller pupils in aged *Gba* KI mice when compared to all other groups (*p* ≤ 0.0031, each). Besides, *Gba* KI mice had smaller pupils than WT mice (*p* = 0.0005), and aged mice had smaller pupils than young mice (*p* = 0.0015). There was no genotype-related effect in the eye size as measured by lens secant length (Fig. 3c; *p* = 0.70).

These data indicate that pupil dilation deficits are not exclusive to pharmacological treatment with the anti-muscarinic compound tropicamide, but are also apparent post-mortem without prior treatment with a pupil dilator. Importantly, differences in eye size are unlikely to account for these genotype-dependent effects.

### Iris pathology in aged *Gba* KI mice

**Figure 4:**
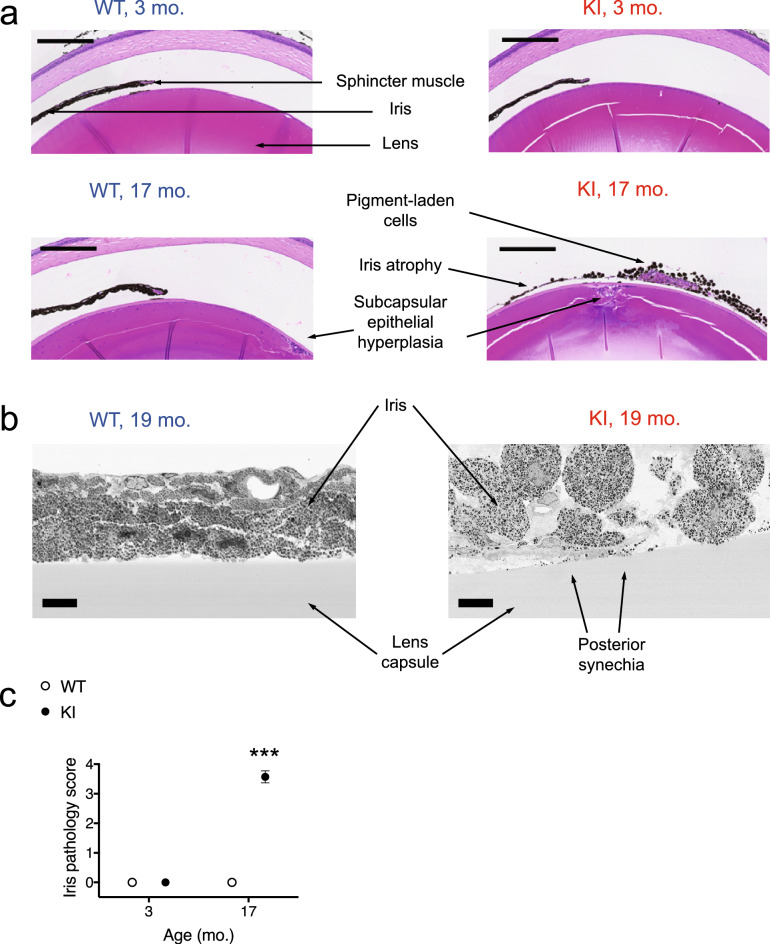
Iris pathology in aged *Gba* KI mice. (**a**) Representative images taken from sagittal histological cross sections through the globe at the level of the optic nerve at higher magnification in 3- versus 17-months old *Gba* WT and KI mice. Note stromal atrophy and pigment dispersion in the iris of aged *Gba* KI mice (bottom right). Posterior synechia were significantly more frequent in aged *Gba* KI mice (Supplementary Table S2). Cataracts and foci of anterior pole subcapsular epithelial hyperplasia occurred more frequently in lenses of aged mice (Supplementary Table S4). The scale bar denotes 200 µm. (**b) **Representative SEM-EM images taken from the iris and the lens capsule in 19-months old *Gba* WT (left panel) and KI (right panel) mice. The scale bar denotes 10 µm. (**c**) An iris pathology score was established based on the aggregation of four individual, categorial, iris-related phenotypes (stromal atrophy, pigment dispersion from the iris, accumulation of pigment-laden cells in the iridocorneal angle (see below), and posterior synechia) yielding a scale with a range from 0 to 4. Iris pathology was pronounced in aged *Gba *KI mice but absent in all other groups (Supplementary table S1).

Next, we examined the microscopic anatomy of the eye using H & E staining. Irises from the aged KI mice showed iris stromal atrophy and adhesions of the posterior iris surface to the anterior surface of the lens (posterior synechia). In young *Gba* KI mice, iris atrophy was occasionally observed, but was limited to the iris stroma, and iris pigment epithelium cells were retained. In contrast, irises from aged *Gba* KI mice showed pigment dispersion, loss of iris pigmented epithelial cells and markedly diminished iris stroma (Fig. 4a, right lower panel). When we scored the presence of posterior synechia in young (3-months old) versus aged (17-months old) *Gba* KI and WT mice, we observed that more than 85% of the aged *Gba* KI mice displayed posterior synechia, but none of the mice in the other groups (Supplementary Table S2, *p* < 0.0001 by Fisher’s exact test). Figure 4b (right panel) shows a SEM-EM picture of posterior synechia in a 19-months old *Gba* KI mouse. Conceivably, posterior synechia may explain the pupil dilation deficits observed in *Gba* KIs.

To better assess the overall severity of iris pathology, an iris pathology score was established consisting of an aggregate score of individual, categorial, iris-related phenotypes, namely stromal atrophy, pigment dispersion from the iris, accumulation of pigment-laden cells in the iridocorneal angle, and posterior synechia. As shown in Fig. 4c, iris pathology was pronounced in aged *Gba* KI mice, but was absent in all other groups (Supplementary Table S2, *p* < 0.0001 by Fisher’s exact test).

Cataracts, often with anterior subcapsular epithelial hyperplasia of the lens, were also observed, but only in aged mice and this phenotype was not genotype-related (Fig. 4, Supplementary Table S4). Cases of corneal opacities as reported for some patients with GD^10^, were not observed in either old or young mice of either genotype.

### Occlusion of iridocorneal angle by pigment-laden cells

**Figure 5:**
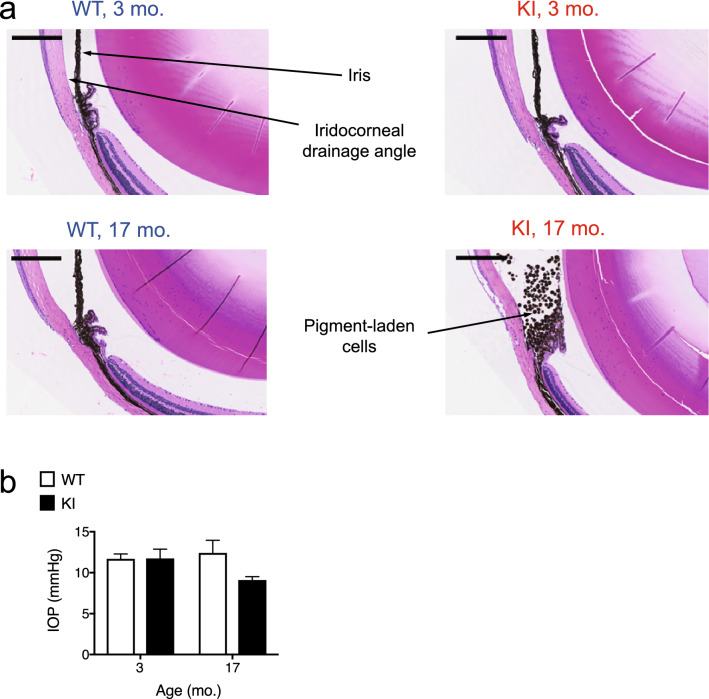
Pigment-laden cells occluding the iridocorneal in aged *Gba* KI mice. (**a**) Representative images taken from sagittal sections through the globe at the level of the optic nerve at higher magnification in 3- versus 17-months old *Gba* KI and WT mice. In aged *Gba* KI mice (bottom right), sheets of pigment-laden cells are occluding the iridocorneal angle, a component of the draining system allowing for the outflow of aqueous humor, consistent with an open-angle glaucoma-like phenotype. This phenotype was significantly more frequent in aged *Gba* KI mice (Supplementary Table S3). (**b**) IOP measures were conducted in anesthetized 2- and 17-months old mice using rebound tonometry. The scale bar denotes 200 µm.

The iridocorneal angle contains the trabecular meshwork and Schlemm’s canal, the ocular drainage structures responsible for aqueous humor outflow. In *Gba* KI mice, the iridocorneal angles contained large numbers of free pigment-laden cells admixed with cellular debris (Fig. 5a). Scoring of this phenotype in 3- versus 17-months old *Gba* KI and WT mice revealed complete penetrance of this phenotype in all aged *Gba* KI mice, but a complete absence in any of the other groups (Supplementary Table S3; *p* < 0.0001 by Fisher’s exact test).

These findings may be relevant in the context of (open angle) glaucoma in mice, where pigment is the main material obstructing the iridocorneal angle^38^. Since the iridocorneal angle itself was not reduced, but appeared blocked, this particular phenotype is reminiscent of secondary open-angle glaucoma.

### Intra-ocular pressure does not differ between anesthetized young and aged *Gba* WT and KI mice

Since the pathology data described above is reminiscent of a glaucoma-like phenotype, we measured IOP via rebound tonometry in anesthetized 2 and 17-month-old *Gba* KI and WT mice (Fig. 5b). However, IOP was not significantly different between these experimental conditions. If anything, there was a mild trend towards an interaction effect of age by genotype (*p* = 0.095), that was driven by numerically lower IOP in aged *Gba* KI mice when compared to the other groups. These data do not support increased IOP in (aged) *Gba* KI mice and—unless IOP is increased in mid-life—rather argue in favor of a glaucoma-like phenotype without prolonged increases in IOP^39^.

### Thinning of retinal layers in aged *Gba* KI mice

**Figure 6:**
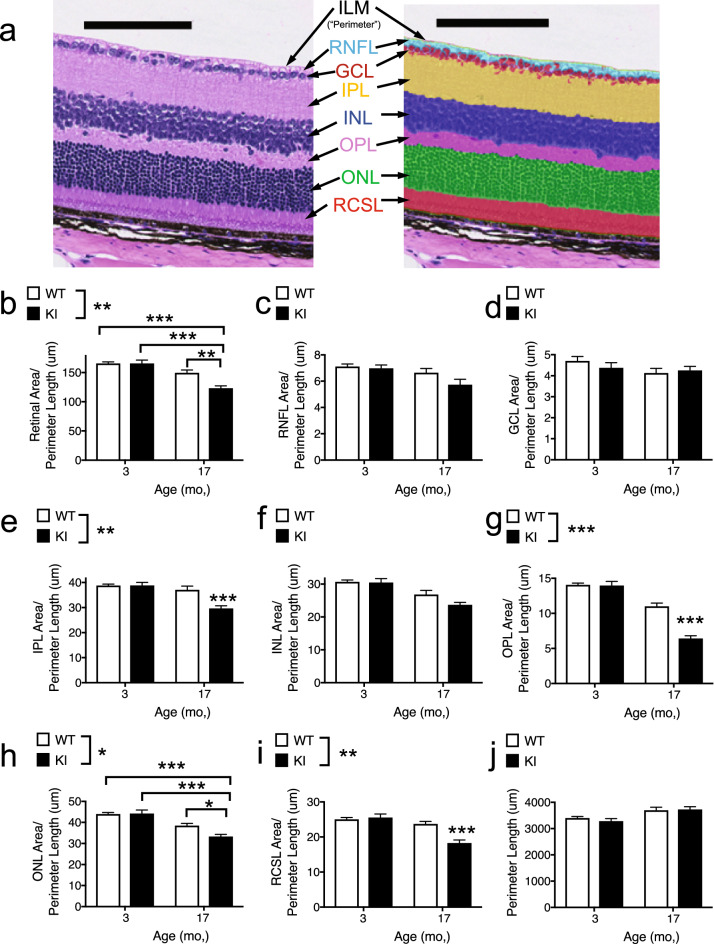
Retinal thinning in aged *Gba* KI mice. (**a**) Example H & E image from a young WT mouse (left) and the same image to which the algorithm for the quantification of retinal layers and sub layers has been applied (right). Area measures of retinal layers were normalized to the approximate length of the ILM which served as “perimeter”, thereby providing a proxy measure for thickness. (**b**–**j**) Area of the total retina (**b**) or the layers indicated (**c**–**i**) normalized to the perimeter length (**j**). Inner limiting membrane (ILM), retinal nerve fiber layer (RNFL), ganglion cell layer (GCL), inner plexiform layer (IPL), inner nuclear layer (INL), outer plexiform layer (OPL), outer nuclear layer (ONL), Rod and cone segments layer (RCSL). The scale bar denotes 100 µm. Asterisks denoting significance levels for main effects of genotype are indicated next to the genotype symbols above the graphs; those for post-hoc tests between individual genotype- and age- combinations are indicated within the graphs. Main effects of age are not indicated. *p < 0.05, **p < 0.01, ***p < 0.001. Values are mean ± SEM.

Measuring the thickness of retinal layers via OCT in aged *Gba* KI mice was not possible due to their pronounced pupil dilation deficits. We therefore measured thickness of the retina and its layers post-mortem from H & E sections in the cohort of 3- versus 17-months old mice (Fig. 6a). The area of the retina, or retinal sub-layers, were normalized to a perimeter line and therefore provide a proxy measure of layer thickness (see “Methods” section).

Most importantly, *Gba* KI mice had ~ 20% thinner retinas compared WT animals at 17 months of age but had similar thickness at 2 months of age, indicating an age-dependent defect (Fig. 6b, *p* = 0.0042). Given these changes in overall retinal thickness, we conducted corresponding analyses for retinal sublayers. There was significant age-dependent thinning in the IPL, OPL, ONL and RCSL sublayers in *Gba* KI mice. Besides, the RNFL and INL layers were numerically thinnest in aged *Gba* KI mice even though these effects did not reach significance. No overt changes between the experimental groups were detected for the GCL (Fig. 6, Supplementary Table S1).

Taken together, these data show that there is progressive retinal thinning in *Gba* KI mice, consistent with retinal thinning in patients with PD and in *GBA1* mutation carriers^16–18^ and that retinal thinning in aged *Gba* KI mice was driven by thinning in multiple retinal layers.

### MALDI-iMS in young and old *Gba* KI mice shows increased levels for GluCer and GluSph in several subregions of the eye

**Figure 7:**
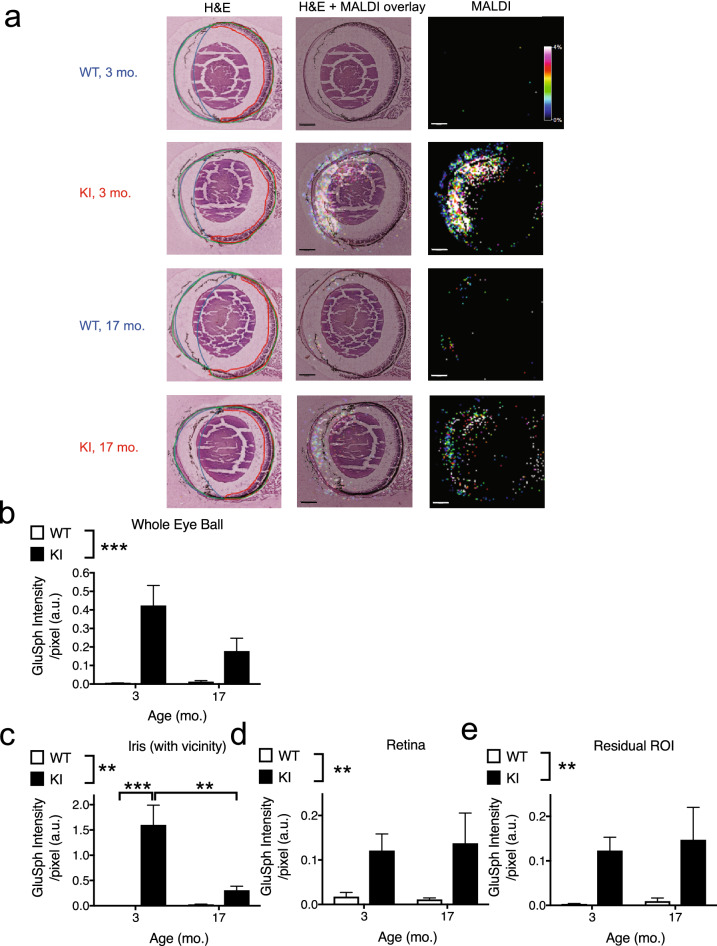
MALDI imaging reveals increased GluSph levels in ocular tissues in *Gba* KI mice. (**a**) The left column shows representative images of H & E sections with an ROI for the entire eyeball (outlined in green). Sub-ROIs within the eye ball included the iris and its vicinity (outlined in blue), the retina (outlined in red), as well as a calculated, residual ROI within the eyeball excluding the other ROIs. The residual ROI mainly corresponds to the lens and the posterior chamber. H & E and MALDI images of GluSph (middle column), and MALDI images of GluSph (right column) are also shown. The scale bar denotes 500 µm. The color scale represents GluSph signal intensity. Quantification of the GluSph levels of the entire eyeball (**b**), the iris (**c**), the retina (**d**), and the ROI of the residual ROI within the eye ball (**e**). Asterisks denoting significance levels for main effects of genotype are indicated next to the genotype symbols above the graphs; those for post-hoc tests between individual genotype- and age-combinations are indicated within the graphs. Main effects of age are not indicated. **p < 0.01, ***p < 0.001. Values are mean ± SEM.

Prompted by the pathology in the retina, the iris, as well as the anterior chamber in the iridocorneal angle in aged *Gba* KI mice, we next examined changes in the abundance of GluCer and GluSph in the eye. Given the limits of resolution provided by the spray-coating of tissues and diffusion of analytes of the MALDI imaging technique, we focused on the whole eye ball, as well as the following sub regions: a crescent-shaped ROI with the corners of the crescent at the ciliary bodies that included the iris as well as sections of the anterior and posterior chambers, the retina, as well as the residual ROI that mainly consisted of the lens as well as the posterior chamber (Fig. 7a, panels to the left).

When compared to WT mice, GluCer in *Gba* KI mice was increased ~ 2.6-fold in the whole eye ball (*p* = 0.0042), ~ 2.4-fold in the iris and its surrounding areas (*p* = 0.021), and ~ 4.9-fold in the residual ROI (*p* = 0.015), but was unaltered in the retina (*p* > 0.2; Figure S2). As reported for the brain and the liver (Fig. 1), the accumulation of GluSph in the eye was more pronounced than that of GluCer.

Specifically, for the whole eyeball *Gba* KI mice had ~ 54 fold increased GluSph levels relative to WT mice (Fig. 7b; *p* < 0.0001). This effect was apparent across both age groups and there was no increase of GluSph with age in *Gba* KI. Given these changes in the entire eyeball, we conducted corresponding analyses for sub regions of the eye ball. For the iris and its surrounding areas, there were changes in GluSph levels with age in *Gba* KI mice (*p* = 0.0030): Interestingly, GluSph levels were highest in young KI mice with ~ 1000-fold higher levels than in young WT mice. GluSph levels in young KI mice were ~ fivefold higher levels than in aged KI mice which may in part reflect the loss of cells in the iris that occurs with age in *Gba* KI mice (Fig. 7c). Besides, GluSph levels were generally higher in KI than WT mice (*p* = 0.0001), and aged mice on average had mildly higher GluSph levels than young mice (*p* = 0.0038). Similar genotype-dependent changes were observed in the retina as well as the residual ROI. In contrast to the iris, there were no age-dependent changes in GluSph levels in these ROIs in either genotype (Fig. 7d,e).

PC (34:1), a phospholipid that is prominently present in most if not all mammalian tissues^40^, did not show genotype or age dependent changes in any of the ocular tissues analyzed (*p* ≥ 0.19, each; Supplementary Table S1, Supplementary Figure S3), indicating the specificity of the changes in GluCer or GluSph in *Gba* KI eyes. In addition, normalizing GluCer and GluSph levels to the integrated abundance of four control lipids (PC 32:0, PC 34:1, PC 36:1, PC 38:6) confirmed the specific accumulation of GluCer and GluSph in the eye compartments (Supplementary Table S1, Supplementary Figures S4, S5). These data show that the observed changes in GluCer and GluSph do not extend indiscriminately to other lipids in *Gba* KI mice.

## Discussion

To the best of our knowledge, this is the first study reporting ocular pathology in a model of GCase impairment. Several ocular phenotypes were discovered in aged *Gba* KI mice. These phenotypes include age-dependent progression of pupil dilation deficits that are likely due to physical adhesions between the iris and the lens, iris atrophy with pigment dispersion and occlusion of the iridocorneal angle, and thinning of multiple retinal layers. MALDI imaging confirmed the presence of elevated GluSph levels in KI mice also in locations where pathology developed with age, indicating that accumulation of GBA substrates could drive the observed ocular phenotypes in aged *Gba* KI mice. These pronounced pathological findings may be particularly valuable as readouts in light of the relatively mild histological phenotypes that have been reported with this *Gba1*^*D409V/D409V*^ KI model to date^19–21^.

Pathological abnormalities and vision dysfunction have been detected in patients with PD and GD, and in other lysosomal storage disorders, including Dannon disease, Fabry disease, and mucopolysaccharidoses^10,12–15,22,41–43^. Specifically, glaucoma has been reported for PD and mucopolysaccharidoses^15,41,42^. Further, there are reports of elevated GluSph levels in ocular tissue of glaucoma patients, suggesting an association of GBA pathway dysfunction and ocular disease even in patients without *GBA1* mutations^44^. However, it is unknown whether an impairment of GCase activity would be sufficient to cause ocular pathology in any patient population. Specifically, it is unclear if the modest changes in GCase activity and the often minor elevations in lipid substrates levels in heterozygous GBA mutation carriers with PD are sufficient to cause such deficits. Yet, we were able to detect glaucoma-like pathology in a genetic mouse model where the *Gba* gene has specifically been mutated resulting in impaired GCase activity. In particular, iris stromal atrophy, loss of iris pigmented epithelial cells and accumulation of pigment-laden cells and cellular debris in the iridocorneal angle was observed in aged *Gba* KI mice. These particular findings are reminiscent of open-angle glaucoma^38^. In addition, MALDI imaging data showed elevated GluSph levels in ocular tissues in KI mice compared to WT mice. These findings are consistent with a pathogenetic role of GBA mutations in the eye.

However, with the exception of retinal thinning (see below) there is no clear relationship between (case) reports of ocular findings in GD, and the pronounced ocular phenotypes detected in this GBA mutation model. For example, while corneal opacities have been observed in a fraction of patients with type 3 GD, we did not detect this phenotype in (aged) *Gba* KI mice. Other pathologies, such as intravitreal bodies which have been detected in some GD patients, could not be investigated in the present study. This is because the vitreous is lost during histological procedures and pupil dilation deficits prevent the *in-vivo* examination of the vitreous in aged *Gba* KI mice. Besides, systematic studies of ocular pathology and function in larger sets of GD patients are critically needed^10^ to better relate findings from animal models to those in humans.

The ocular pathology detected here is reminiscent of pigment dispersion syndrome (PDS), a disease in humans, and in mouse models, where pigmented cells detach from the iris and accumulate at the iridocorneal angle, leading to glaucoma in 35 to 50% of patients^45–49^. We therefore propose that loss of GCase activity results in elevated GluSph lipids in the eye which may then lead to detachment of pigmented cells from the iris, accumulation of the cells in the iridocorneal angle. In future studies, this hypothesis may be tested by injecting synthetic GluSph directly into the eye to determine if the pathology can be recapitulated and by treating the mice with brain-penetrant compounds which selectively reduce GluSph levels in the eye of *Gba* KI mice.

As with any animal model of human disease, it is also important to note some findings that are less consistent with the human disease phenotype of glaucoma. For example, despite significant thinning of the IPL, we did not detect widespread pathology in the inner retinal layers, including the GCL, even though this is typical in glaucoma. Second, while increased IOP is often, but not necessarily, a symptom of glaucoma, we did not detect genotype- or age-dependent changes in IOP. While there are several possible explanations for this result, the most parsimonious explanation is that the magnitude of the open angle obstruction in aged *Gba* KI mice might not be sufficient to increase IOP. This is consistent with findings that substantial alterations at the iridocorneal angle in mice with closed angle glaucoma are needed in order to elevate IOP^50^.

Retinal degeneration has also been found in both GD and PD patients^16–18^. We therefore investigated whether impairment of GCase activity might be sufficient to induce retinal degeneration in *Gba* KI mice. Here, we report age-dependent thinning of several layers of the retina in *Gba* KI mice. Interestingly, retinal thinning occurred in several layers rich in neuronal cells or axons: First the RCSL, i.e. the layer of the light-sensing rod and cone segments which are part of specialized cells with synapses and axons; second the ONL where the cell bodies of the rods and cones are located; third the OPL, where rods and cone cells interface with horizontal cells; forth the IPL, i.e. the interface between axons of bipolar cells and ganglion cells. While retinal thinning was most widespread in the outer retinal layers, we did detect significant thinning in the IPL of the inner retinal layer. This is in line with findings of pronounced thinning of the IPL in human PD patients^17^. Besides, the RNFL and INL of the inner retinal layer were numerically thinnest in aged KI mice, even though these effects did not reach significance. Overall, these data indicate that loss of GCase activity and elevations in GluSph lipids can lead to retinal degeneration, likely accompanied by impairment in visuospatial acuity.

We have also tested mice for other known mutations with retinal phenotypes, including *Rd1*, *Rd10* and *Rd12*. All tested mice were negative for these mutations. Thus, none of these mutations is a passenger mutation of the *Gba* knock-in and none any of these mutations contributes to the phenotypes described here. One potential caveat of our retinal findings is that the *Gba* KI was bred, in part, on the C57BL/6N background for which retinal lesions linked to the *Rd8* mutation in the *Crb1* gene have been reported^25^. Indeed, all the *Gba* WT and KI mice used for the data analyses of the main paper were homozygous for the *Rd8* mutation. This shows that the *Rd8* mutation is not a passenger mutation of the *Gba* knock-in^28–31^. Yet, while these experimental groups were identical for the *Rd8* mutation, and we have not detected overt retinal lesions of the type described by^25^, we cannot exclude that an interaction of the *Rd8* mutation with the *Gba* KI and possibly age contributes to the retinal thinning reported here.

If degeneration of the retina reflects neurodegeneration in the brain, the easy access of retinal tissues via imaging methods like OCT makes the eye a prime target for biomarker approaches aimed to diagnose neurodegenerative disease, track disease progression, or track the effects of therapeutic interventions. For example, retinal thinning was detected by OCT in *GBA1* mutation carriers at risk of developing neurodegeneration^16^. Furthermore, retinal thinning in PD patients has been correlated with the loss of dopaminergic cells in the substantia nigra^17^. It would be intriguing to calculate the corresponding correlations between retinal thinning and brain pathology in the present mouse model. However, such studies are less promising given the challenges to detect the small magnitude of brain pathology reported to date^19–21^. Yet, our characterization of the *Gba* KI mice demonstrates the utility of the model in validating retinal thinning as a biomarker for treatment effects of novel GBA-directed therapies. While the changes in ocular histopathology and lipid levels reported here focused on the comparison of young (3 months) versus aged (17 months old) adult mice in order to maximize the age effect, future studies may be valuable to closely track these changes over time. The near-linear progression of pupil dilation deficits documented here (Figure S1) where significant differences between the genotypes were detected as early as four (pupil-to-limbus-diameter ratio) and seven months of age (pupil diameter) may be valuable in guiding these studies such that the earliest time points of histopathological and lipid alterations can be identified and applied to biomarker studies.

In summary, the ocular phenotypes described in this model may be useful to elucidate the consequences of GCase impairment, to develop translatable biomarkers for PD and for GD, and to ultimately test the effects of disease-modifying therapeutics aimed to normalize impaired GCase signaling.

## Supplementary Information

Supplementary Information
