# Supplementary material for: Ocular phenotypes in a mouse model of impaired glucocerebrosidase activity
Source: Sci Rep. 2021 Mar 16;11:6079. doi: 10.1038/s41598-021-85528-4 (PMC7971029; doi:10.1038/s41598-021-85528-4)
Supplement: Supplementary file 1 — Supplementary Information [file 41598_2021_85528_MOESM1_ESM.docx]

**SUPPLEMENTS**

**Ocular phenotypes in a mouse model of impaired glucocerebrosidase activity**

Martin Weber^1^*^&^, Sang-Won Min^1&^, Tom Truong^2^, Jeffrey Hung^3^, Stephanie Dale^4^, Mike Reichelt^3^, Savita Ubhayakar^4^, Carol Cain-Hom^5^, Miriam Baca^3^, Zhiyu Jiang^1^, Qingling Li^6^, Robert Brendza^1^, Han Lin^1^, Chung Kung^5^, William F. Forrest^7^, Cristine Quiason-Huynh^4^, Wendy Sandoval^6^, Buyun Chen^4^, Yuzhong Deng^4^, Amy Easton^1^, Oded Foreman^3#^, Abdoulaye Sene^2#^, Baris Bingol^1#*^

^&^ equal contribution; ^#^ equal contribution

Departments of 1. Neuroscience, 2. Translational Immunology, 3. Research Pathology, 4. Drug Metabolism and Pharmacokinetics, 5. Transgenic Technology, 6. Microchemistry, Proteomics and Lipidomics, 7. OMNI Bioinformatics, Genentech Inc., 1 DNA Way, South San Francisco, CA 94080

*Correspondence should be addressed to:

Dr. Martin Weber, Email: weber.martin@gene.com or

Dr. Baris Bingol; Email: bingol.baris@gene.com

**SUPPLEMENTARY MATERIALS AND METHODS**

**Genotyping for murine mutations linked to retinal degeneration phenotypes**

The sequence of the gBlocks (Integrated DNA Technologies) are listed below:

*Rd1* wild-type gBlock: TTAATTCTGGGGCGCATGGTCTACCCACCCTTCCTAATTTTTCTCACGCTTCCTCCTGCCTTCTTTCATTCATTCCCACCTTACCCTTTTCTTCCCCCTCCCACCCACCTTCAGGGAATCTTCAAAGCAGTGCCTACACGTAGCCCTGAGCCTGAGGCAGGTTCTCTCCTTTCTATTGCCCTGATCCACACCAAAGTTGCTCTGTGGTGTTGCTCTGCGGTAAGATGAAGTTGCAGAGTTGTCCTTAATTCTATTCCCAGGCCTACCCATGTCCTACAGCCCCTCTCCAAGGTTTATAGTCACTCTGTGGCCTCAAAGATACATCCTTGGTGGCACATCATGTCTACTACTGTTATGATGATTCTGTGACCTGCAGGTTGTGCAGGTTTGTATTGATCCCAGACTGAGGGAAAAGGAGTAACCGTCAAAGTCAGCAAAATCCATATTTC

*Rd1* mutant gBlock:

GTAAACAGCAAGAGGCTTTATTGGGAACACGGGTACCCGGCGACTCAGTCTGTCGGAGGACTGGCGCGCGAGTGTGGGGTTTTTACCCTTTTTATAGGGCTGGGGAGCAAAAAGCGTGGGTACAGAAGCGAGAAGCGAGCTGAGTGGTTAGTTCAAATCAAGGCTTGGGGTATTTCCCGGTCATTTGGGGAACCTGAAACTGAGGTGGGACTTTTTCAGAAACTGTTGCTGGTTCCGCTTTATCTGGGTGCCATCTGTTCACGTAGCCCTGAGCCTGAGGCAGGTTCTCTCCTTTCTATTGCCCTGATCCACACCAAAGTTGCTCTGTGGTGTTGCTCTGCGGTAAGATGAAGTTGCAGAGTTGTCCTTAATTCTATTCCCAGGCCTACCCATGTCCTACAGCCCCTCTCCAAGGTTTATAGTCACTCTGTGGCCTCAAAGATACATCCTTGGTGGCACATCATGTCTACTACTGTTATGATGATTCTGTGACCTGCAGGTTGTGCAGGTTTGTATTGATCCCAGACTGAGGGAAAAGGAGTAACCGTCA

*Rd8* wild-type gBlock:

TGACACCTAGTTTTAATATTTTGATCCATGCTCCTAAAGTCTATCCTTGTACTTTTTAAACCACCAAATTGCATAAGAAAAACTGATGTTCTGTCTATAGGTAAGCTGACAAATAACAAATAGGCACATAGAAAATCTAGTAAGTAGTACCACCTGATATCTCACTTTGCTGCAGGCAGGATTCCCCTCTGGCTCACTGGCAGTCTCCTCCGATGTGGGCCAGGGCTCTTTGAAGTTGGATCTGAGCCTTTCTATCACCTGTTTGATGGACAAGCCCTTTTGCACAAGTTTGACTTCAAGGAGGGCCATGTCACATACCACAGAAGGTAAGTCCATGACTGACCTCACCAGAGTAGAGCCTATCTCAGTTCTTCTCCTGTTTGAACTTTCACCTTCATGAACCCAGAGAAAACCTCTAAACACATTTCTTTCTTCTTTCTTCATGGTCTAGCCATGTCTGTATATTTGCACAGCCACCTGAGAATGTTAAAATGAGATAATGGGGTTCAAGAGCTCTG

*Rd8* mutant gBlock:

GACAATACAGACATCTATGTGGGTGACCAATCTGTTGACAATCCGAAAGGCCTGCAGGGCTGTCTGAGCACAATAGAGATTGGAGGCATATATCTTTCTTACTTTGAAAATCTACATGGTTTCCCTGGTAAGCCTCAGGAAGAGCAATTTCTCAAAGTTTCTACAAATATGGTACTTACTGGCTGTTTGCCATCAAATGCCTGCCACTCCAGCCCCTGTTTGCATGGAGGAAACTGTGAAGACAGCTACAGTTCTTATGGTGTGCCTGTCTCTCGGGATGGTCAGGGACACACTGTGAAATCAACATTGATGAGTGCTTTTCTAGCCCCTGTATCCATGGCAACTGCTCTGATGGAGTTGCAGCCTACCACTGCAGGTGTGAGCCTGGATACACCGGTGTGAACTGTGAGGTGGATGTAGACAATTGCAAGAGTCATCAGTGTGCAAATGGGGCCACCTGTGTTCCTGAAGCTCAT

*Rd10* wild-type gBlock:

GGGGCCAGTGAGAACAAGGAACAAGGGCTCTGAGACCAACAAGATAGAAGTGGAGAAATAGGTATAACAGACTCTAATGCAAGCAGTATGAGAGGCTTGGATAGGCTCTGATATGGTGCTGTGTAGGCTCATATGTGGATCTCAGAACCCACATGTACTCTGCTCCCCAGGTCTTGGTGCGCTTTCTATTCTCTGTCAGCAAAGCCTATCGAAGAATCACCTACCACAACTGGCGCCACGGCTTCAATGTAGCCCAGACCATGTTTACCCTACTCATGGTACGTATGTAAATTGGATGGGCTAGATGAATCAGAGGGCTGGGGCAAGGACCACAGCTAACTATCTTCTGGCCCAAGGATGCCAATTGTGTGTATCCAGTCCTAGCAATGAGTGGAAGGGACCTGGGTGGGCAAAG

*Rd10* mutant gBlock:

GGCCAGTGAGAACAAGGAACAAGGGCTCTGAGACCAACAAGATAGAAGTGGAGAAATAGGTATAACAGACTCTAATGCAAGCAGTATGAGAGGCTTGGATAGGCTCTGATATGGTGCTGTGTAGGCTCATATGTGGATCTCAGAACCCACATGTACTCTGCTCCCCAGGTCTTGGTGCGCTTTCTATTCTCTGTCAGCAAAGCCTATCGAAGAATCACCTACCACAACTGGTGCCACGGCTTCAATGTAGCCCAGACCATGTTTACCCTACTCATGGTACGTATGTAAATTGGATGGGCTAGATGAATCAGAGGGCTGGGGCAAGGACCACAGCTAACTATCTTCTGGCCCAAGGATGCCAATTGTGTGTATCCAGTCCTAGCAATGAGTGGAAGGGACCTGGGT

*Rd12* wild-type gBlock:

TGACACCTAGTTTTAATATTTTGATCCATGCTCCTAAAGTCTATCCTTGTACTTTTTAAACCACCAAATTGCATAAGAAAAACTGATGTTCTGTCTATAGGTAAGCTGACAAATAACAAATAGGCACATAGAAAATCTAGTAAGTAGTACCACCTGATATCTCACTTTGCTGCAGGCAGGATTCCCCTCTGGCTCACTGGCAGTCTCCTCCGATGTGGGCCAGGGCTCTTTGAAGTTGGATCTGAGCCTTTCTATCACCTGTTTGATGGACAAGCCCTTTTGCACAAGTTTGACTTCAAGGAGGGCCATGTCACATACCACAGAAGGTAAGTCCATGACTGACCTCACCAGAGTAGAGCCTATCTCAGTTCTTCTCCTGTTTGAACTTTCACCTTCATGAACCCAGAGAAAACCTCTAAACACATTTCTTTCTTCTTTCTTCATGGTCTAGCCATGTCTGTATATTTGCACAGCCACCTGAGAATGTTAAAATGAGATAATGGGGTTCAAGAGCTCTG

*Rd12* mutant gBlock:

TGACACCTAGTTTTAATATTTTGATCCATGCTCCTAAAGTCTATCCTTGTACTTTTTAAACCACCAAATTGCATAAGAAAAACTGATGTTCTGTCTATAGGTAAGCTGACAAATAACAAATAGGCACATAGAAAATCTAGTAAGTAGTACCACCTGATATCTCACTTTGCTGCAGGCAGGATTCCCCTCTGGCTCACTGGCAGTCTCCTCTGATGTGGGCCAGGGCTCTTTGAAGTTGGATCTGAGCCTTTCTATCACCTGTTTGATGGACAAGCCCTTTTGCACAAGTTTGACTTCAAGGAGGGCCATGTCACATACCACAGAAGGTAAGTCCATGACTGACCTCACCAGAGTAGAGCCTATCTCAGTTCTTCTCCTGTTTGAACTTTCACCTTCATGAACCCAGAGAAAACCTCTAAACACATTTCTTTCTTCTTTCTTCATGGTCTAGCCATGTCTGTATATTTGCACAGCCACCTGAGAATGTTAAAATGAGATAATGGGGTTCAAGAGCTCTG

**Pupil dilation measures**

In order to better map the exact time course of the age-related changes for pupil dilation, data from five cohorts of mice from which pupil dilation data was obtained was used. This included female mice as well as mice where the genotypes for *Rd1*, *Rd8*, *Rd10* and *Rd12* were not known (n = 121: 36 M, *Gba* WT/WT; 30 M, *Gba* KI/KI; 26 F, *Gba* WT/WT; 29 F, *Gba* KI/KI). The ages on test days ranged from 2 to 17 months of age (2.3 months of age on average in the youngest age group to 16.8 months in the oldest age group). Testing occurred in a manner counterbalanced by genotype and - when multiple ages were tested simultaneously - age to avoid confounding effects of time of day, or testing sequence between groups. In a small fraction of recordings (7 of a total of 121 mice) where sufficient anesthesia was not achieved with ketamine/xylazine, mice were briefly exposed to 4% of isoflurane in addition. Otherwise measures were conducted as outlined in the method sections of the main paper. Mice from two of these cohorts (n=38) were used for repeated testing. Data were therefore analyzed with two factorial mixed-effects ANOVAs, where genotype was the between-subject factor and age the within-subjects factor. *Gba* KI/KI mice are also referred to as “KI” mice and *Gba* WT/WT mice as “WT” mice for short.

**Matrix-assisted laser desorption ionization imaging mass spectrometry (MALDI-IMS)**

MALDI experiments were conducted as outlined in the main paper

**SUPPLEMENTARY RESULTS**

**Pupil dilation deficits in response to the anti-muscarinic compound tropicamide in aged *Gba* KI mice**

*Gba* KI mice showed consistent reductions in pupil diameter following tropicamide treatment (**Supplementary Figure S1a**, p<0.0001), and aged mice had smaller pupils when compared to young mice (p<0.0001). Importantly, the genotype effect was further aggravated with age (p<0.0001) with significantly smaller pupil diameters in *Gba* KIs starting at the age of seven months with progression to almost 5-fold smaller pupils at 17 months (**Supplementary Figure S1a, Supplementary Table S1**). The reduction in pupil size was not due an overall change in eye size between genotypes since normalization of pupil diameter with limbus diameter (pupil to limbus ratios, **Supplementary Figure S1b, Supplementary Table S1**) showed similar results with significant decrease in the ratio in *Gba* KI mice starting at four months and worsening with age in both genotypes (**Supplementary Figure S1b**, p<0.0001). These data demonstrate an age-dependent progression of pupil dilation defects in *Gba* KI mice.


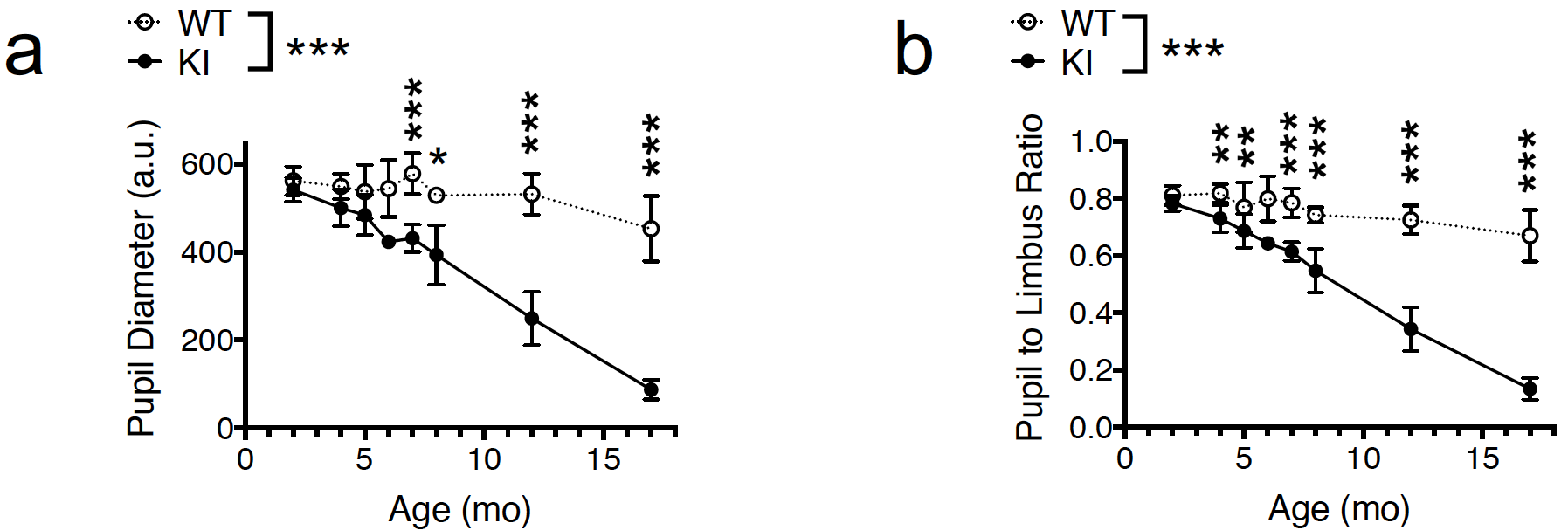


**Supplementary Figure S1. Pupil dilation deficits in response to tropicamide progress with age in *Gba* KI mice**

(**a**) Pupil diameter following tropicamide treatment in *Gba* KI and WT mice of various ages. (**b**) Pupil to limbus diameter ratio measures were calculated to normalize pupil measures relative to limbus diameter, a proxy measure fore eye size. Asterisks denoting significance levels for main effects of genotype are indicated next to the genotype symbols above the graphs; those for post-hoc tests between genotypes of the same age are indicated within the graphs. Main effects of age are not indicated. *p < 0.05, **p < 0.01, ***p < 0.001. Values are mean ± SEM.

**MALDI-iMS in young and old *Gba* KI mice shows increased levels for GluCer in several subregions of the eye**

When compared to WT mice, GluCer in *Gba* KI mice was increased in the whole eye ball (p=0.0042), the iris and its surrounding areas (p=0.021), and in the residual ROI (p=0.015), but was unaltered in the retina (p>0.2; **Figure S2**).


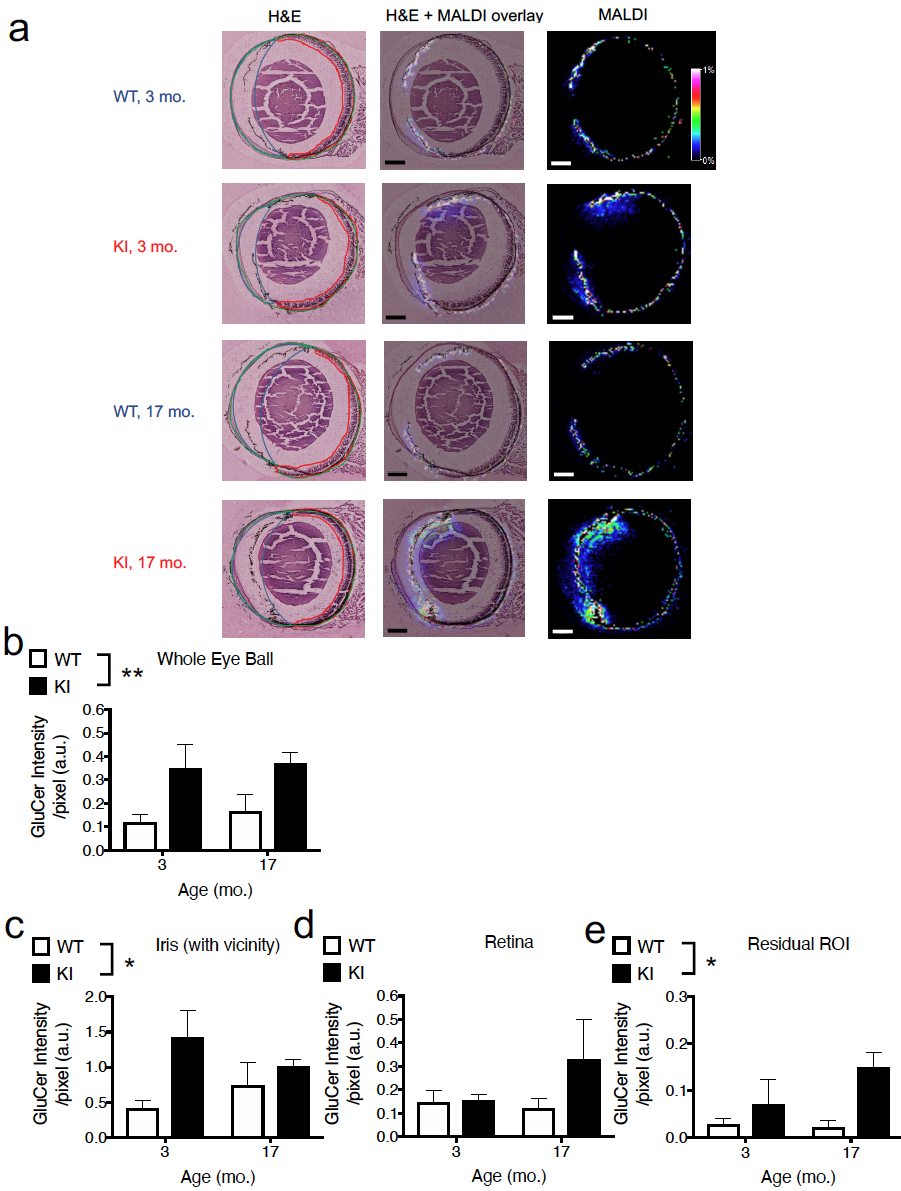


**Supplementary Figure S2: MALDI imaging reveals increased GluCer levels in ocular tissues in *Gba* KI** **mice**

(**a**) The left column shows representative images of H & E sections with an ROI for the entire eyeball (outlined in green). Sub-ROIs within the eye ball included the iris and its vicinity (outlined in blue), the retina (outlined in red), as well as a calculated, residual ROI within the eyeball excluding the other sub-ROIs. The residual ROI mainly corresponds to the lens and the posterior chamber. H & E and MALDI images of GluCer (middle column), and MALDI images of GluCer (right column) are also shown. The scale bar denotes 500 μm. The color scale represents GluCer signal intensity. Quantification of the GluCer levels of the entire eyeball (**b**), the iris (**c**), the retina (**d**), and the residual ROI within the eye ball (**e**). GluCer levels were increased in *Gba* KI mice for the whole eye ball, the iris with vicinity and the residual area, but not the retina. *p < 0.05, **p < 0.01. Values are mean ± SEM.

**MALDI-iMS in young and old *Gba* KI mice shows unaltered levels for PC 34:1 in the eye**

PC (34:1), a phospholipid that is prominently present in most if not all mammalian tissues served as a negative control for the metabolic effects of *Gba* KI. There were no significant effects of genotype, age, or genotype by age on PC (34:1) levels in any of the ocular tissues analyzed (p≥0.19, each; **Supplementary Table S1, Supplementary Figure S3**). This shows that the observed changes in GluSph do not extend indiscriminately to other lipids in *Gba* KI mice.

**Supplementary Figure S3: MALDI imaging reveals normal PC (34:1) levels in ocular tissues in *Gba* KI** **mice**

(**a**) The left column shows representative images of H & E sections with an ROI for the entire eyeball (outlined in green). Sub-ROIs within the eye ball included the iris and its vicinity (outlined in blue), the retina (outlined in red), as well as a calculated, residual ROI within the eyeball excluding the other sub-ROIs. The residual ROI mainly corresponds to the lens and the posterior chamber. H & E and MALDI images of PC (34:1; middle column), and MALDI images of PC (34:1; right column) are also shown. The scale bar denotes 500 μm. The color scale represents PC (34:1) signal intensity. Quantification of the PC (34:1) levels of the entire eyeball (**b**), the iris (**c**), the retina (**d**), and the residual ROI within the eye ball (**e**). PC (34:1) levels were similar between genotypes and ages in 3 and 17 months old *Gba* KI and WT mice in all regions analyzed.

**MALDI-iMS in young and old *Gba* KI mice shows increased levels for GluCer and GluSph in subregions of the eye relative to control lipids in young (3 mo.) WT mice**

Normalizing GluCer and GluSph levels to the integrated abundance of four control lipids (PC 32:0, PC 34:1, PC 36:1, PC 38:6) confirmed the specific accumulation of GluCer and GluSph in compartments of the eye (**Supplementary Table S1, Supplementary Figures S4, S5**).


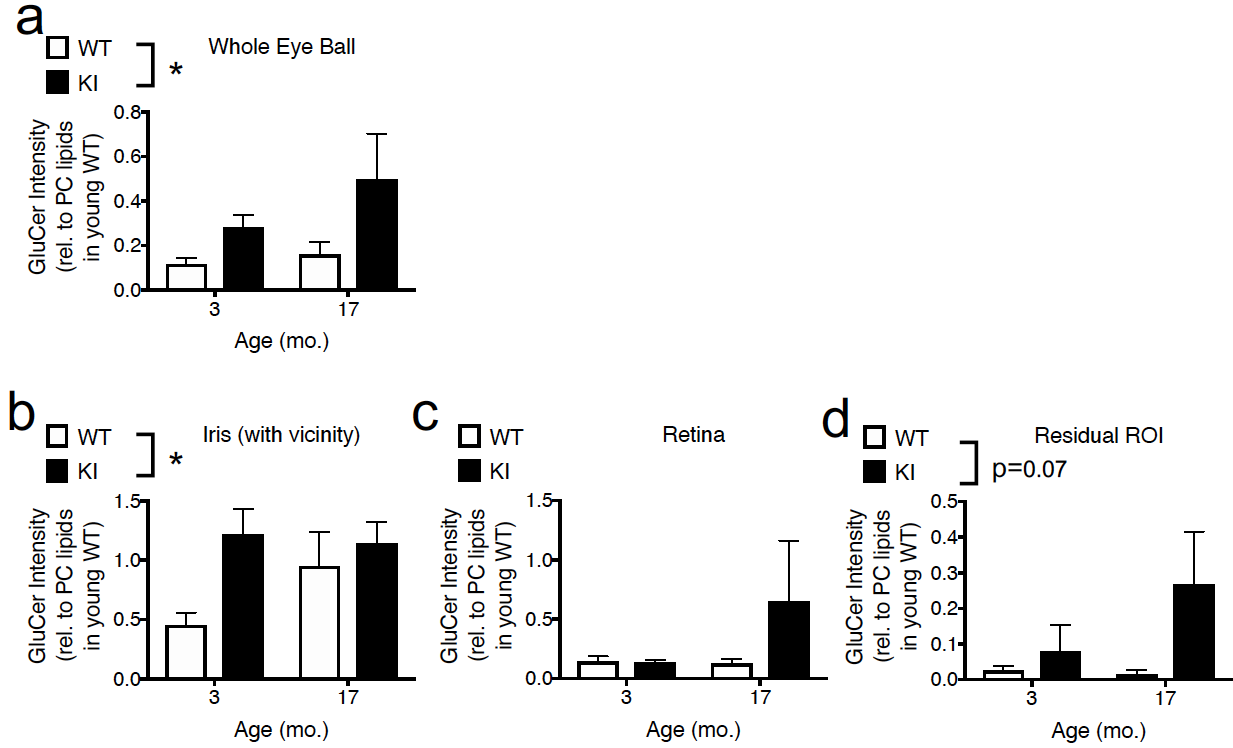


**Figure S4: GluCer levels in ocular tissues in *Gba* KI mice relative to PC lipids in 3 months old WT mice**

To exclude the possibility of unspecific effects on lipid levels in general, GluCer levels were normalized relative to an aggregate measure of 4 control lipids (PC 32:0, PC 34:1, PC 36:1, PC 38:6) in 3 months old WT mice. Quantification of the normalized GluCer levels of the entire eyeball (**b**), the iris (**c**), the retina (**d**), and the ROI of the residual ROI within the eye ball (**e**). Asterisks denoting significance levels for main effects of genotype are indicated next to the genotype symbols above the graphs; those for post-hoc tests between individual genotype- and age- combinations are indicated within the graphs.*p < 0.05. Values are mean ± SEM.

**Figure S5: GluSph levels in ocular tissues in *Gba* KI mice relative to PC lipids in 3 months old WT mice**

To exclude the possibility of unspecific effects on lipid levels in general, GluSph levels were normalized relative to an aggregate measure of 4 control lipids (PC 32:0, PC 34:1, PC 36:1, PC 38:6) in 3 months old WT mice. Quantification of the normalized GluSph levels of the entire eyeball (**b**), the iris (**c**), the retina (**d**), and the ROI of the residual ROI within the eye ball (**e**). Asterisks denoting significance levels for main effects of genotype are indicated next to the genotype symbols above the graphs; those for post-hoc tests between individual genotype- and age- combinations are indicated within the graphs. Main effects of age are not indicated.**p < 0.01, ***p < 0.001. Values are mean ± SEM.

| **Figure or table panel** | **Assay** | **Statistical test, findings** | **Post-hoc test results**  (if genotype x age effects were significant) | **No. of subjects** |
| --- | --- | --- | --- | --- |
| Figure 1a | GCase activity  (forebrain) | ANOVA  **Genotype**: F(1,25)=1120,3, p<0.0001; KI<WT;  **Age**: (F(1,25)=1.6, p=0.22;  **Genotype x age**: F(1,25)<1, p=0.80 | n.a. | aged KI = 7;  aged WT = 7;  young KI = 6;  young WT = 9 |
| Figure 1b | GluCer (forebrain) | ANOVA  **Genotype**: F(1,25)=12.1, p=0.0018, KI>WT;  **Age**: F(1,25)<1, p=0.35;  **Genotype x age**: F(1,25)<1, p=0.83 | n.a. | aged KI = 7;  aged WT = 7;  young KI = 6;  young WT = 9 |
| Figure 1c | GluSph (forebrain) | ANOVA  **Genotype**: F(1,25)=496.2, p<0.0001, KI>WT;  **Age**: F(1,25)<1, p=0.41;  **Genotype x age**: F(1,25)<1, p=0.41 | n.a. | aged KI = 7;  aged WT = 7;  young KI = 6;  young WT = 9 |
| Figure 1d | GalCer (forebrain) | ANOVA  **Genotype**: F(1,25)<1, p=0.96;  **Age**: F(1,25)<1, p=0.76;  **Genotype x age**: F(1,25)=1.0, p=0.32 | n.a. | aged KI = 7;  aged WT = 7;  young KI = 6;  young WT = 9 |
| Figure 1e | GalSph (forebrain) | ANOVA  **Genotype**: F(1,25)<1, p=0.38;  **Age**: F(1,25)=72.5, p<0.0001, a>y;  **Age x genotype**: F(1,25)<1, p=0.41 | n.a. | aged KI = 7;  aged WT = 7;  young KI = 6;  young WT = 9 |
| Figure 1f | GluCer (liver) | ANOVA  **Genotype**: F(1,18)=44.9, p<0.0001, KI>WT;  **Age**: F(1,18)<1, p=0.76;  **Age x genotype**: F(1,18)=1.9, p=0.18 | n.a. | aged KI = 5;  aged WT = 5;  young KI = 5;  young WT = 7 |
| Figure 1g | GluSph (liver) | ANOVA  **Genotype**: F(1,18)=754.1, p<0.0001, KI>WT;  **Age**: F(1,18)<1, p=0.65;  **Age x genotype**: F(1,18)<1, p=0.65 | n.a. | aged KI = 5;  aged WT = 5;  young KI = 5;  young WT = 7 |
| Figure 2b | Pupil diameter (in-life, tropicamide) | ANOVA  **Genotype**: F(1,25)=197.0, p<0.0001, KI<WT;  **Age**: F(1,25)=380.1, p<0.0001; a<y; **Genotype x age**: F(1,25)=133.0, p<0.0001 | Tukey’s post-hoc tests  **Aged KI:** < all other groups (p≤0.0001); **Young KI:** vs. young WT (p>0.30), > aged WT (p=0.0057); **Aged WT:** < young WT (p<0.0001) | aged KI = 7;  aged WT = 7;  young KI = 6;  young WT = 9 |
| Figure 2c | Pupil to limbus diameter ratio (in-life, tropicamide) | ANOVA  **Genotype**: F(1,25)=260.7, p<0.0001, KI<WT;  **Age**: F(1,25)=502.4, p<0.0001;  **Genotype x age**: F(1,25)=184.1, p<0.0001 | Tukey’s post-hoc tests  **Aged KI:** < all other groups (p≤0.0001); **Young KI:** vs. young WT (p>0.28), > aged WT (p=0.0015); **Aged WT:** < young WT (p<0.0001) | aged KI = 7;  aged WT = 7;  young KI = 6;  young WT = 9 |
| Figure 3b | Pupil secant length (H & E stains) | ANOVA  **Genotype**: F(1,23)=16.5, p=0.0005, KI<WT;  **Age**: F(1,23)=9.0, p=0.0063; a<y;  **Genotype x age**: F(1,23)=6.8, p=0.016 | Tukey’s post-hoc tests  **Aged KI:** < aged WT (p=0.0009), < young KI (p=0.0031), < young WT (p<0.0001);  **all other comparisons:** p>0.69 | aged KI = 7;  aged WT = 5;  young KI = 6;  young WT = 9 |
| Figure 3c | Lens secant length (H & E stains) | ANOVA  **Genotype**: F(1,23)<1, p=0.70;  **Age**: F(1,23)=132.0, p<0.0001, a>y; **Genotype x Age**: F(1,23)=1.9, p=0.18 | n.a. | aged KI = 7;  aged WT = 5;  young KI = 6;  young WT = 9 |
| Figure 4c | Iris pathology score (H & E stains) | Fisher’s exact test  (across all 4 groups and 5 score levels)  p<0.0001 | n.a.  (difference between groups is exclusively driven by aged KI mice) | aged KI = 7;  aged WT = 5;  young KI = 6;  young WT = 9 |
| Figure 5b | IOP (in-life) | ANOVA  **Genotype**: F(1,25)=2.8, p=0.108;  **Age**: F(1,25)<1;  **Genotype x Age**: F(1,25)=3.0, p=0.095 | n.a. | aged KI = 8;  aged WT = 7;  young KI = 6;  young WT = 8 |
| Figure 6b | Total retina | ANOVA  **Genotype**: F(1,24)=9.8, p=0.0045; KI<WT;  **Age**: F(1,24)=49.2, p<0.0001, a<y;  **Genotype x age**: F(1,24)=10.0, p=0.0042 | Tukey’s post-hoc tests  **Aged KI:** < aged WT (p=0.0012), < young KI (p<0.0001), < young WT (p<0.0001);  **Young KI:** vs. young WT (p>0.99), vs aged WT (p=0.073); **Aged WT:** vs. young WT (p=0.046) | aged KI = 7;  aged WT = 6;  young KI = 6;  young WT = 9 |
| Figure 6c | RNFL | ANOVA  **Genotype**: F(1,24)= 3.0, p=0.096;  **Age**: F(1,24)=8.0, p=0.0091; a<y;  **Genotype x age**: F(1,24)=1.7, p=0.21 | n.a. | aged KI = 7;  aged WT = 6;  young KI = 6;  young WT = 9 |
| Figure 6d | GCL | ANOVA  **Genotype**: F(1,24)<1, p=0.66;  **Age**: F(1,24)=2.4, p=0.13;  **Genotype x age**: F(1,24)<1, p=0.33 | n.a. | aged KI = 7;  aged WT = 6;  young KI = 6;  young WT = 9 |
| Figure 6e | IPL | ANOVA  **Genotype**: F(1,24)=12.2, p=0.0019, KI<WT;  **Age**: F(1,24)=26.5, p<0.0001, a<y;  **Genotype x age**: F(1,24)=12.5, p=0.0017 | Tukey’s post-hoc tests  **Aged KI:** < aged WT (p=0.0003), < young KI (p<0.0001), < young WT (p<0.0001);  **all other comparisons** (p≥0.64) | aged KI = 7;  aged WT = 6;  young KI = 6;  young WT = 9 |
| Figure 6f | INL | ANOVA  **Genotype**: F(1,24)=3.3, p=0.083;  **Age**: F(1,24)=33.5, p<0.0001, a<y;  **Genotype x age**: F(1,24)=2.6, p=0.12 | n.a. | aged KI = 7;  aged WT = 6;  young KI = 6;  young WT = 9 |
| Figure 6g | OPL | ANOVA  **Genotype**: F(1,24)=31.9, p<0.0001, KI<WT;  **Age**: F(1,24)=164.5, p<0.0001; a<y; **Genotype x age**: F(1,24)=29.4, p<0.0001 | Tukey’s post-hoc tests  **Aged KI:** < all other groups (p<0.0001, each); **Young KI:** vs. young WT (p>0.99), > aged WT (p=0.0004); **Aged WT:** < young WT (p<0.0001) | aged KI = 7;  aged WT = 6;  young KI = 6;  young WT = 9 |
| Figure 6h | ONL | ANOVA  **Genotype**: F(1,24)=4.9, p=0.036, KI<WT;  **Age**: F(1,24)=54.2, p<0.0001, a<y;  **Genotype x age**: F(1,24)=5.9 p=0.023 | Tukey’s post-hoc tests  **Aged KI:** < aged WT (p=0.019), < young KI (p<0.0001), < young WT (p<0.0001); **Young KI:** vs. young WT (p>0.99), > aged WT (p=0.012); **Aged WT:** vs. young WT (p=0.0076) | aged KI = 7;  aged WT = 6;  young KI = 6;  young WT = 9 |
| Figure 6i | RCSL | ANOVA  **Genotype**: F(1,24)=10.3, p=0.0037, KI<WT;  **Age**: F(1,24)=31.2, p<0.0001, a<y;  **Genotype x age**: F(1,24)=14.8, p=0.0008 | Tukey’s post-hoc tests  **Aged KI:** < aged WT (p=0.0003), < young KI (p<0.0001), < young WT (p<0.0001); **all other comparisons** (p≥0.41) | aged KI = 7;  aged WT = 6;  young KI = 6;  young WT = 9 |
| Figure 6j | Perimeter length | ANOVA  **Genotype**: F(1,24)<1, p=0.64;  **Age**: F(1,24)=16.2, p=0.0005, a>y;  **Genotype x age**: F(1,24)<1, p=0.42 | n.a. | aged KI = 7;  aged WT = 6;  young KI = 6;  young WT = 9 |
| Figure 7b | MALDI for GluSph (whole eye ball) | **Genotype**: F(1,17)=22.9, p<0.0001, KI>WT;  **Age**: F(1,17)=3.8, p=0.068;  **Genotype x age**: F(1,17)=4.3, p=0.053 | n.a. | aged KI = 6;  aged WT = 5;  young KI = 5;  young WT = 5 |
| Figure 7c | MALDI for GluSph (iris with vicinity) | ANOVA  **Genotype**: F(1,17)=24.6, p=0.0001, KI>WT;  **Age**: F(1,17)=11.2, p=0.0038, a>y;  **Genotype x age**: F(1,17)=12.0, p=0.0030 | Tukey’s post-hoc tests  **Young KI**: > young WT (p<0.0001), > aged KI (p=0.0010); > aged WT (p=0.0001);  **all other comparisons** p>0.65 | aged KI = 6;  aged WT = 5;  young KI = 5;  young WT = 5 |
| Figure 7d | MALDI for GluSph (retina) | ANOVA  **Genotype**: F(1,17)=9.5, p=0.0068, KI>WT;  **Age**: F(1,17)<1, p=0.89;  **Genotype x age**: F(1,17)<1, p=0.76 | n.a. | aged KI = 6;  aged WT = 5;  young KI = 5;  young WT = 5 |
| Figure 7e | MALDI for GluSph (Residual ROI: Lens & posterior chamber) | ANOVA  **Genotype**: F(1,17)=11.8, p=0.0031, KI>WT;  **Age**: F(1,17)<1, p=0.68;  **Genotype x age**: F(1,17)<1, p=0.82 | n.a. | aged KI = 6;  aged WT = 5;  young KI = 5;  young WT = 5 |
| Supplementary Figure S1a | Pupil diameter (in-life, tropicamide) | ANOVA  **Genotype**: F(1,84)=230.7, p<0.0001, KI<WT;  **Age**: F(7,129)=106.2, p<0.0001; **Genotype x age**: F(7,129)=45.1, p<0.0001 | Tukey’s post-hoc tests  **2 mo.**: p>0.99;  **4 mo.**: p=0.25;  **5 mo.**: p=0.054;  **6 mo.**: p=0.056;  **7 mo.**: p<0.0001, KI<WT;  **8 mo.**: p=0.031, KI<WT;  **12 mo.**: p<0.0001, KI<WT;  **17 mo.**: p<0.0001, KI<WT | KI = 59  WT = 62 |
| Supplementary Figure S1b | Pupil to limbus diameter ratio (in-life, tropicamide) | ANOVA  **Genotype**: F(1,67)=311.9, p<0.0001, KI<WT;  **Age**: F(7,134)=141.5, p<0.0001;  **Genotype x age**: F(7,134)=59.1, p<0.0001 | Tukey’s post-hoc tests  **2 mo.**: p>0.99;  **4 mo.**: p=0.0050, KI<WT;  **5 mo.**: p=0.0032, KI<WT;  **6 mo.**: p=0.051,  **7 mo.**: p=0.0001, KI<WT;  **8 mo.**: p=0.0071, KI<WT;  **12 mo.**: p<0.0001, KI<WT;  **17 mo.**: p<0.0001, KI<WT | KI = 59  WT = 62 |
| Supplementary Figure S2b | MALDI for GluCer (whole eye ball) | ANOVA  **Genotype**: F(1,17)=10.9, p=0.0042, KI>WT;  **Age**: F(1,17)<1, p=0.61;  **Genotype x age**: F(1,17)<1, p=0.86 | n.a. | aged KI = 6;  aged WT = 5;  young KI = 5;  young WT = 5 |
| Supplementary Figure S2c | MALDI for GluCer (iris with vicinity) | ANOVA  **Genotype**: F(1,17)=6.4, p=0.021, KI>WT;  **Age**: F(1,17)<1, p=0.86;  **Genotype x age**: F(1,17)=2.2, p=0.16 | n.a. | aged KI = 6;  aged WT = 5;  young KI = 5;  young WT = 5 |
| Supplementary Figure S2d | MALDI for GluCer (retina) | ANOVA  **Genotype**: F(1,17)=1.51, p=0.24;  **Age**: F(1,17) <1, p=0.42;  **Genotype x age**: F(1,17)=1.24, p=0.28 | n.a. | aged KI = 6;  aged WT = 5;  young KI = 5;  young WT = 5 |
| Supplementary Figure S2e | MALDI for GluCer (residual ROI of lens & posterior chamber) | ANOVA  **Genotype**: F(1,17)=7.4, p=0.015, KI>WT;  **Age**: F(1,17)=1.36, p=0.26;  **Genotype x age**: F(1,17)=1.86, p=0.19 | n.a. | aged KI = 6;  aged WT = 5;  young KI = 5;  young WT = 5 |
| Supplementary Figure S3b | MALDI for PC (34:1; whole eye ball) | ANOVA  **Genotype**: F(1,17)<1, p=0.75;  **Age**: F(1,17)<1, p=0.64;  **Genotype x age**: F(1,17)<1, p=0.79 | n.a. | aged KI = 6;  aged WT = 5;  young KI = 5;  young WT = 5 |
| Supplementary Figure S3c | MALDI for PC (34:1; iris with vicinity) | ANOVA  **Genotype**: F(1,17)<1, p=0.53;  **Age**: F(1,17)= 1.83, p=0.19;  **Genotype x age**: F(1,17)<1, p=0.57 | n.a. | aged KI = 6;  aged WT = 5;  young KI = 5;  young WT = 5 |
| Supplementary Figure S3d | MALDI for PC (34:1; retina) | ANOVA  **Genotype**: F(1,17)<1, p=0.44;  **Age**: F(1,17)<1, p=0.66;  **Genotype x age**: F(1,17)=1.57, p=0.23 | n.a. | aged KI = 6;  aged WT = 5;  young KI = 5;  young WT = 5 |
| Supplementary Figure S3e | MALDI for PC (34:1; residual ROI of lens & posterior chamber) | ANOVA  **Genotype**: F(1,17)<1, p=0.79;  **Age**: F(1,17)<1, p=0.56;  **Genotype x age**: F(1,17)<1, p=0.97 | n.a. | aged KI = 6;  aged WT = 5;  young KI = 5;  young WT = 5 |
| Supplementary Figure S4a | MALDI for GluCer (whole eye ball; values normalized to PC lipids in 3 mo. old mice) | ANOVA  **Genotype**: F(1,17)=5.6, p=0.030, KI>WT;  **Age**: F(1,17)=1.47, p=0.24;  **Genotype x age**: F(1,17)<1, p=0.43 | n.a. | aged KI = 6;  aged WT = 5;  young KI = 5;  young WT = 5 |
| Supplementary Figure S4b | MALDI for GluCer (iris with vicinity; values normalized to PC lipids in 3 mo. old mice) | ANOVA  **Genotype**: F(1,17)=5.7, p=0.029, KI>WT;  **Age**: F(1,17)=1.09, p=0.31;  **Genotype x age**: F(1,17)=2.07, p=0.17 | n.a. | aged KI = 6;  aged WT = 5;  young KI = 5;  young WT = 5 |
| Supplementary Figure S4c | MALDI for GluCer (retina; values normalized to PC lipids in 3 mo. old mice) | ANOVA  **Genotype**: F(1,17)=1.11, p=0.31;  **Age**: F(1,17) =1.01, p=0.33;  **Genotype x age**: F(1,17)=1.14, p=0.30 | n.a. | aged KI = 6;  aged WT = 5;  young KI = 5;  young WT = 5 |
| Supplementary Figure S4d | MALDI for GluCer (residual ROI of lens & posterior chamber; values normalized to PC lipids in 3 mo. old mice) | ANOVA  **Genotype**: F(1,17)=3.7, p=0.071;  **Age**: F(1,17)=1.20, p=0.29;  **Genotype x age**: F(1,17)=1.54, p=0.23 | n.a. | aged KI = 6;  aged WT = 5;  young KI = 5;  young WT = 5 |
| Supplementary Figure S5a | MALDI for GluSph (whole eye ball; values normalized to PC lipids in 3 mo. old mice) | ANOVA  **Genotype**: F(1,17)=8.7, p=0.0091, KI>WT;  **Age**: F(1,17)<1, p=0.97;  **Genotype x age**: F(1,17)<1, p=0.91 | n.a. | aged KI = 6;  aged WT = 5;  young KI = 5;  young WT = 5 |
| Supplementary Figure S5b | MALDI for GluSph (iris with vicinity; values normalized to PC lipids in 3 mo. old mice) | ANOVA  **Genotype**: F(1,17)=51.4, p<0.0001, KI>WT;  **Age**: F(1,17)=14.0, p=0.0016, y>a;  **Genotype x age**: F(1,17)=16.6, p=0.0.0008 | Tukey’s post-hoc tests  **Young KI**: > young WT (p<0.0001), > aged KI (p=0.0002); > aged WT (p<0.0001);  **all other comparisons** p>0.09 | aged KI = 6;  aged WT = 5;  young KI = 5;  young WT = 5 |
| Supplementary Figure S5c | MALDI for GluSph (retina; values normalized to PC lipids in 3 mo. old mice) | ANOVA  **Genotype**: F(1,17)=3.1, p=0.098;  **Age**: F(1,17) <1, p=0.46;  **Genotype x age**: F(1,17)<1, p=0.42 | n.a. | aged KI = 6;  aged WT = 5;  young KI = 5;  young WT = 5 |
| Supplementary Figure S5d | MALDI for GluSph (residual ROI of lens & posterior chamber; values normalized to PC lipids in 3 mo. old mice) | ANOVA  **Genotype**: F(1,17)=2.35, p=0.14;  **Age**: F(1,17)<1, p=36;  **Genotype x age**: F(1,17)<1, p=0.38 | n.a. | aged KI = 6;  aged WT = 5;  young KI = 5;  young WT = 5 |
| Supplementary Table S2 | Posterior synechia (H & E stains) | Fisher’s exact test  (across all 4 groups)  p<0.0001 | n.a.  (difference between groups is exclusively driven by aged KI mice, see Supplementary Table S2) | aged KI = 7;  aged WT = 5;  young KI = 6;  young WT = 9 |
| Supplementary Table S3 | Occlusion of iridocorneal angle (H & E stains) | Fisher’s exact test  (across all 4 groups)  p<0.0001 | n.a.  (difference between groups is exclusively driven by aged KI mice, see Supplementary Table S3) | aged KI = 7;  aged WT = 5;  young KI = 6;  young WT = 9 |
| Supplementary Table S4 | Cataracts (H & E stains) | Fisher’s exact test  (across all 4 groups)  p=0.0029 | n.a.  (Fisher’s exact tests:  **Aged KI** vs. **aged WT** and **young KI** vs. **young WT**: p>0.99, each. Thus, the differences between groups is driven by age and not by a genotype-related effect) | aged KI = 7;  aged WT = 5;  young KI = 6;  young WT = 9 |

**Supplementary Table S1. Summary of statistical tests results**.

The table lists the number of the figure panel or table (1^st^ column from left), the assay (2^nd^ column from left), the results of the ANOVA or Fisher’s exact tests (3^rd^ column from left), the results of the post-hoc tests (4^th^ column from left) when interaction effects of genotype by age were significant, and the sample sizes (column to the very right) of the corresponding data. “<” and “>” refer to “less than” and “greater than”, respectively. Abbreviations: KI = KI/KI mice, WT = WT/WT mice, a = aged (17-months old) mice , y = young ( 2 or 3-months old) mice.

| **Phenotype: Posterior synechia** | | | | | | | | | |  |
| --- | --- | --- | --- | --- | --- | --- | --- | --- | --- | --- |
|  |  | **Age (months)** | | | | | | | |  |
|  |  | **3** | | | | | **17** | | |  |
| **# of mice** |  |  | **WT** | **KI** | **Σ** |  | **WT** | **KI** | **Σ** |  |
| without phenotype |  |  | 9 | 6 | 15 |  | 5 | 1 | 6 |  |
| with phenotype |  |  | 0 | 0 | 0 |  | 0 | 6 | 6 |  |
| Σ |  |  | 9 | 6 | 15 |  | 5 | 7 | 12 |  |
| **% with phenotype** |  |  | **0** | **0** |  |  | **0** | **85.7** |  |  |

**Supplementary Table S2. Posterior synechia is common in aged *Gba* KI mice.**

The table lists the number of mice from both ages (3- vs. 17-months old) and genotypes (*Gba* WT vs. KI) that did or did not display the phenotype of posterior synechia. The % of mice with posterior synechia is shown in the grey cells. There were significant differences between groups (p<0.0001 by Fisher’s exact test) which were exclusively driven by aged *Gba* KI mice.

| **Phenotype: Pigment-laden cells near the iridocorneal angle** | | | | | | | | | |  |
| --- | --- | --- | --- | --- | --- | --- | --- | --- | --- | --- |
|  |  | **Age (months)** | | | | | | | |  |
|  |  | **3** | | | | | **17** | | |  |
| **# of mice** |  |  | **WT** | **KI** | **Σ** |  | **WT** | **KI** | **Σ** |  |
| without phenotype |  |  | 9 | 6 | 15 |  | 5 | 0 | 5 |  |
| with phenotype |  |  | 0 | 0 | 0 |  | 0 | 7 | 7 |  |
| Σ |  |  | 9 | 6 | 15 |  | 5 | 7 | 12 |  |
| **% with phenotype** |  |  | **0** | **0** |  |  | **0** | **100** |  |  |

**Supplementary Table S3. Pigment-laden cells in proximity to the iridocorneal angle in aged *Gba* KI mice**.

The table lists the number of mice from both ages (3- vs. 17-months old) and genotypes (*Gba* WT vs. KI) that did or did not display the phenotype of pigment-laden cells in proximity to the iridocorneal angle. The % of mice with pigment-laden cells close to the iridocorneal angle is shown in grey cells. There were significant differences between groups which were exclusively driven by aged *Gba* KI mice.

| **Phenotype: Presence of cataract(s)** | | | | | | | | | |  |
| --- | --- | --- | --- | --- | --- | --- | --- | --- | --- | --- |
|  |  | **Age (months)** | | | | | | | |  |
|  |  | **3** | | | | | **17** | | |  |
| **# of mice** |  |  | **WT** | **KI** | **Σ** |  | **WT** | **KI** | **Σ** |  |
| without phenotype |  |  | 9 | 6 | 15 |  | 2 | 3 | 5 |  |
| with phenotype |  |  | 0 | 0 | 0 |  | 3 | 4 | 7 |  |
| Σ |  |  | 9 | 6 | 15 |  | 5 | 7 | 12 |  |
| **% with phenotype** |  |  | **0** | **0** |  |  | **60** | **57.1** |  |  |

**Supplementary Table S4. Cataracts are common in aged mice.**

The table lists the number of mice from both ages (3- vs. 17-months old) and genotypes (*Gba* WT vs. KI) that did or did not display the phenotype of (a) cataract(s) in the lens. The % of mice with cataract(s) is shown in grey cells. There were significant differences between groups (p=0.0029 by Fisher’s exact test) which were exclusively driven by age.
